# Use of MSAP Markers to Analyse the Effects of Salt Stress on DNA Methylation in Rapeseed (*Brassica napus* var. *oleifera*)

**DOI:** 10.1371/journal.pone.0075597

**Published:** 2013-09-23

**Authors:** Gianpiero Marconi, Roberta Pace, Alessandra Traini, Lorenzo Raggi, Stanley Lutts, Marialuisa Chiusano, Marcello Guiducci, Mario Falcinelli, Paolo Benincasa, Emidio Albertini

**Affiliations:** 1 Department of Applied Biology, University of Perugia, Perugia, Italy; 2 Department of Agricultural and Environmental Science, University of Perugia, Perugia, Italy; 3 Department of Soil, Plant, Environmental and Animal Production Sciences, University of Naples Federico II, Naples, Italy; 4 Groupe de Recherche en Physiologie végétale, Earth and Life Institute-Agronomy, Université Catholique de Louvain, Louvain-la-Neuve, Belgium; Cankiri Karatekin University, Turkey

## Abstract

Excessive soil salinity is a major ecological and agronomical problem, the adverse effects of which are becoming a serious issue in regions where saline water is used for irrigation. Plants can employ regulatory strategies, such as DNA methylation, to enable relatively rapid adaptation to new conditions. In this regard, cytosine methylation might play an integral role in the regulation of gene expression at both the transcriptional and post-transcriptional levels. Rapeseed, which is the most important oilseed crop in Europe, is classified as being tolerant of salinity, although cultivars can vary substantially in their levels of tolerance. In this study, the Methylation Sensitive Amplified Polymorphism (MSAP) approach was used to assess the extent of cytosine methylation under salinity stress in salinity-tolerant (Exagone) and salinity-sensitive (Toccata) rapeseed cultivars. Our data show that salinity affected the level of DNA methylation. In particular methylation decreased in Exagone and increased in Toccata. Nineteen DNA fragments showing polymorphisms related to differences in methylation were sequenced. In particular, two of these were highly similar to genes involved in stress responses (Lacerata and trehalose-6-phosphatase synthase S4) and were chosen to further characterization. Bisulfite sequencing and quantitative RT-PCR analysis of selected MSAP loci showed that cytosine methylation changes under salinity as well as gene expression varied. In particular, our data show that salinity stress influences the expression of the two stress-related genes. Moreover, we quantified the level of trehalose in Exagone shoots and found that it was correlated to *TPS*4 expression and, therefore, to DNA methylation. In conclusion, we found that salinity could induce genome-wide changes in DNA methylation status, and that these changes, when averaged across different genotypes and developmental stages, accounted for 16.8% of the total site-specific methylation differences in the rapeseed genome, as detected by MSAP analysis.

## Introduction

Excessive salt accumulation in soil is a major ecological and agronomical problem [1], the adverse effects of which are becoming a serious issue in regions where saline water is used for irrigation [2]. Globally, more than 45 million hectares of irrigated land have been damaged by salt, and 1.5 million hectares are taken out of production each year as a result of high levels of soil salinity [3,4]. The detrimental effects of high salinity on plants can be observed at the whole-plant level as plant death or a decrease in productivity. During the onset and development of salt stress, major processes such as photosynthesis, protein synthesis, and energy and lipid metabolism, are affected [5]. Long-term exposure to salinity induces ionic stress, which leads to premature senescence of adult leaves, and thus to reduced rates of photosynthesis [4,6]. In fact, ionic stress causes symptoms of toxicity, such as chlorosis and necrosis, due to high Na^+^ levels, which affects plants by disrupting protein synthesis and interfering with enzyme activity [7–9]. A high concentration of Na^+^ also reduces growth and inhibits cell division and expansion [10].

During their evolution, plants have evolved several mechanisms either to exclude salt from their cells or to tolerate its presence within the cells. These mechanisms include the synthesis and accumulation of compatible solutes to avoid cell dehydration and maintain root water uptake, the fine regulation of the uptake and distribution of water to plant tissues by aquaporins, the reduction of oxidative damage through improved antioxidant capacity, and the maintenance of photosynthesis at a level adequate for plant growth [1]. In general, molecular responses are activated because plants perceive stress as it occurs and relay that information through a signal transduction pathway, which leads to physiological changes or to changes in the expression of specific genes [11].

Plants can employ regulatory strategies, such as DNA methylation, to enable relatively rapid adaptation to new conditions [12]. In this regard, cytosine methylation may play an integral role in the regulation of gene expression at both the transcriptional and post-transcriptional levels [13,14]. Specifically, DNA methylation involves the addition of a methyl group to 5-methylcytosine or N6-methyladenine [15–21]. Changes dependent on the methylation of cytosine residues in genomic DNA and, specifically, those in the promoter sequences of specific genes, play a pivotal role in the regulation of genome functions [19,22]. Stress-induced changes in methylation might account, at least in part, for how plants adapt to stress. Environmental stimuli, such as aluminium, heavy metals, and water stress, alter cytosine methylation at specific loci throughout the genome [23], which affects the expression of specific genes. A study on *Ribes* germplasm suggested that temporal changes in DNA methylation status might cause epigenetic modulation of gene activity during sucrose-mediated acclimation to cold temperatures. In particular, whereas DNA methylation was induced in the cryotolerant genotypes tested, demethylation was evident in cryosensitive genotypes [24]. Osmotic stresses induced transient DNA hypermethylation at two heterochromatic loci in tobacco cell-suspension cultures [25], whereas DNA hypermethylation was induced by drought stress of pea root tips [26]. In tobacco, DNA demethylation in the coding sequence of a gene induced by aluminium, paraquat, salt, and cold stress was correlated with expression of the gene [27]. Steward et al. [28] reported that genome-wide demethylation occurred in maize root tissues when seedlings were exposed to cold stress. The screening of genomic DNA identified a fragment (designated ZmMI1) that was demethylated upon exposure to cold. A particularly noteworthy finding was that ZmMI1 was expressed only under cold stress.

The effects of salt stress on cytosine methylation have been investigated in several crops [29-32]. In particular, Lu et al. [29] investigated the extent and pattern of DNA methylation changes under saline conditions in *Brassica napus* using the methylation-sensitive amplified polymorphism (MSAP) technique. This technique is based on the use of the isoschizomers *Hpa*II and *Msp*I, which differ in their sensitivity to methylation in their recognition sites. Both enzymes recognise the tetranucleotide sequence 5′-CCGG-3′, but their actions are affected by the methylation state of the first or second cytosine residue. Although *Hpa*II is inactive when either or both of the cytosines are fully methylated (both strands methylated), it cleaves the hemi-methylated sequence (only one strand methylated), whereas *Msp*I cleaves hemi- or fully methylated C5mCGG, but not 5mCCGG [33]. The MSAP technique has also been applied to assess the extent and pattern of cytosine methylation in the genomes of several other species [30-32,34–41]. In their paper, Lu et al. [29] showed that the locations in the rapeseed genome of alterations in DNA methylation induced by salt stress are random. They also identified two methylation polymorphic fragments that share high homology with *Brassica oleracea* genes that encode an ethylene responsive element binding factor-related protein and *Arabidopsis* copia-like retrotransposon, but did not perform further investigations. Wang et al. [31] investigated changes in DNA methylation in salt-stressed roots and leaves at various developmental stages of two rice genotypes with different degrees of salt tolerance. They found that DNA methylation changes occur throughout the entire rice genome in response to salt stress. In particular, their results showed that decreases in DNA methylation in roots at the seedling stage induced by salt stress were greater in the salt-sensitive than in the salt-insensitive genotype. They stated that their results may point to gene demethylation in seedling roots as an active response to salt stress. Upon comparing four rice genotypes with different levels of tolerance to salt stress using MSAP, Karan et al. [32] did not observe any methylation pattern specific to salt-tolerant or salt-sensitive genotypes in roots or shoots under salt stress; they suggested that the evolution of natural genetic variability for salinity tolerance in germplasm may have been independent of the extent and pattern of DNA methylation in rice.

Rapeseed (*Brassica napus* var. *oleifera* Del.), a natural allotetraploid (AACC; 2n = 4× = 38) derived from *B* *. rapa* L. (AA; 2n = 2× = 20) and *B. oleracea* L (CC; 2n = 2× = 18), is the most important oilseed crop in Europe [42], particularly in Mediterranean regions. Although *B. napus* is classified as being tolerant of salinity, substantial variability in the level of tolerance between cultivars has been reported [43]. Recent findings demonstrated that the tolerance of rapeseed to salinity during germination and initial seedling growth is strongly dependent on the genotype [44]. Two cultivars (the salinity-tolerant cultivar Exagone from Monsanto and the salinity-sensitive cultivar Toccata from Maisadour Semences) exhibit contrasting behaviour to salt and water stress, while having similar imbibition kinetics when incubated in distilled water [43,44]. The authors attributed the superior salinity tolerance of Exagone to less damage to the DNA of Exagone than to that of Toccata, and a reduced requirement for DNA replication during radical elongation (which results primarily from cell elongation rather than from cell division) when the two cultivars were exposed to salt stress. Moreover, it was proposed that the higher concentration of soluble sugars found in salt-stressed tissues of Exagone than in salt-stressed tissues of Toccata might led to osmotic adjustment, and therefore to the maintenance of turgor even when the external concentration of NaCl was high [44].

The hypothesis of this study was to verify if the different responses of cultivars to salt stress are related to differences in methylation profile and, therefore, to the activation/inactivation of specific genes. With this aim on mind, we analysed the extent and pattern of cytosine methylation in the presence and absence of salinity stress in both the salinity-tolerant cultivar Exagone and the salinity-sensitive cultivar Toccata using the MSAP technique.

## Results

### Extent and pattern of DNA methylation under control conditions and salinity stress

**Figure 1:**
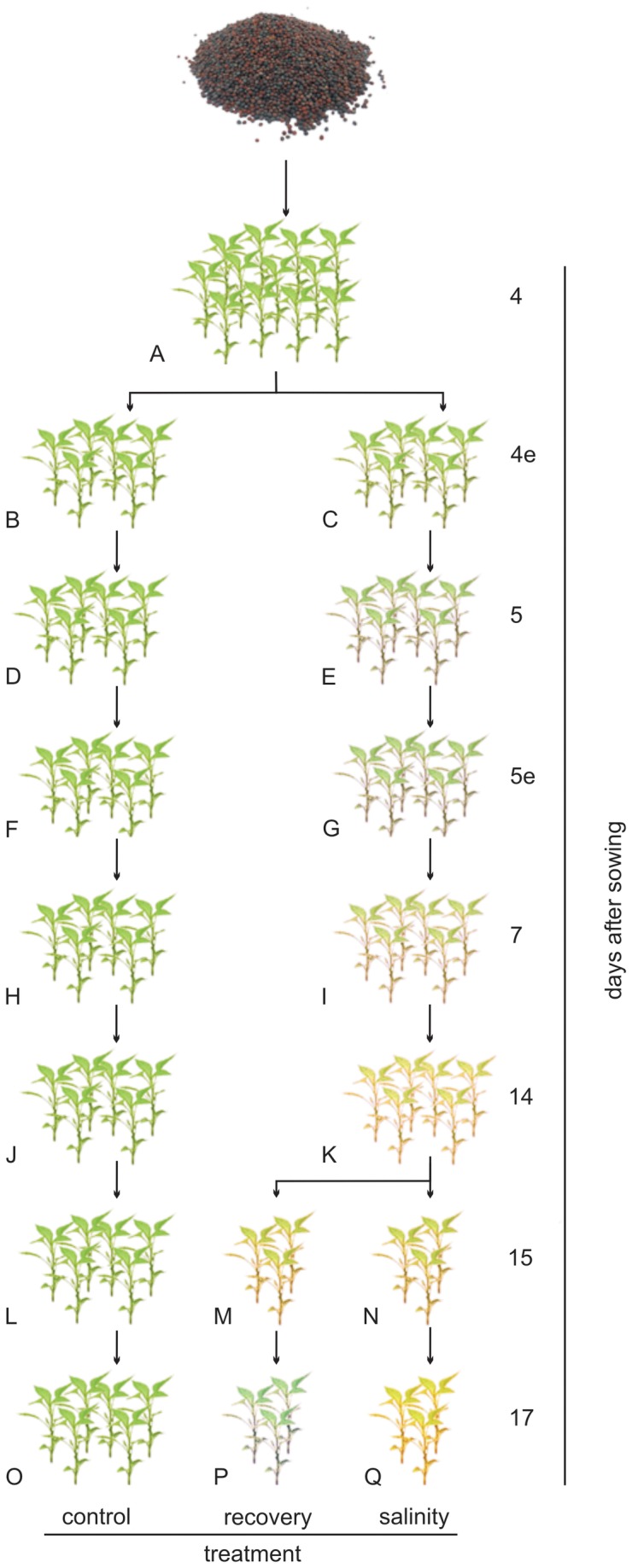
Stages of collection of seedlings. Seeds were sterilised in 0.1% NaClO, incubated in distilled water (0 mmol/L), and placed on moistened filter paper. Four days after sowing (4 DAS), seedlings (A) were split and grown in boxes containing distilled water (control; B, D, F, H, J, L, O) or NaCl solution (100 mmol/L; C, E, G, I, K). Moreover, at 14 DAS samples grown under stressed conditions (K) were split: half were kept under stressed conditions (N and Q) and the other half were grown under control conditions (recovery; M and P). All samples were collected at 8 am, except for samples indicated with the letter e (evening) which were collected at 8 pm. Samples 4e, 5 and 5e were used for qRT-PCR and not for MSAP analysis.

Fifteen primer combinations (Table S1) were used to assay cytosine methylation at 5’-CCGG-3’ sequences in two rapeseed cultivars found to be either tolerant (Exagone) or sensitive (Toccata) to salt stress during different seedling developmental stages. Samples were collected from plants grown under stress and control conditions at 4, 7, and 14 days after sowing (DAS). Moreover, samples after recovery from salinity stress (samples subjected to salinity stress until 14 DAS and then grown under non-stress conditions for one or three days) were collected at 15 and 17 DAS for inclusion in the analysis (Figure 1).

A total of 772 and 783 clear and reproducible bands (Figure S1) were amplified from Exagone and Toccata samples, respectively. Under the control conditions, the total methylation of CCGG sequences averaged 46.58% in Exagone and 41.02% in Toccata. The extent of DNA methylation ranged from 45.85% (7 DAS) to 47.67% (4 DAS) in Exagone and from 39.71% (14 DAS) to 43.55% (4 DAS) in Toccata samples (Table 1). Salinity stress decreased the percentage of total methylated bands in tolerant Exagone (avg of 38.27%), but increased it in sensitive Toccata (avg of 48.66%), when levels of DNA methylation were compared with those in the respective unstressed control. These findings indicated opposite effects of salinity stress on DNA methylation in genotypes of *B. napus* with different levels of tolerance to salinity stress (Table S2). The fully methylated loci were always more abundant than the hemi-methylated ones (Table 1).

In Exagone, level of methylation (avg of 49.67%) and banding pattern after recovery from stressed conditions resembled those observed under control conditions. However, the relative abundances of fully and hemi-methylated bands changed during the recovery period. In fact, whereas there was an evident increase in fully methylated bands in samples after recovery from salinity stress (avg of 46.69%) compared with the proportion in salinity-stressed ones (avg of 35.75%), the levels of hemi-methylated bands remained similar (avg of 2.98% and 2.52% in samples after recovery and salinity-stressed samples, respectively). Methylation of Toccata in recovered samples remained at a level similar to that of salinity-stressed ones (avg of 50.25% vs. 48.66%), even though the relative abundance of fully and hemi-methylated bands changed. In fact, there was evidence of a decrease in the number of hemi-methylated bands (from an avg of 5.16% to 3.44%) and an increase of fully methylated bands (from an avg of 43.48% to 46.8%) in recovered samples compared with salinity-stressed ones.

**Table 1:** DNA methylation changes at seedling developmental stages of salt-tolerant Exagone and salt-sensitive Toccata under three water conditions.

	**Exagone**										
	**H_2_O**						**NaCl**			**Recovery**	
**MSAP band type**	**4**	**7**	**14**	**15**	**17**		**7**	**14**		**15**	**17**
I	404	418	414	410	416		474	479		389	388
II	12	11	7	8	9		20	19		22	24
III	144	142	148	148	145		138	134		124	128
IV	212	201	203	206	202		140	140		237	232
Tot. Amplified bands	772	772	772	772	772		772	772		772	772
Tot. methylated bands	368	354	358	362	356		298	293		383	384
Fully methylated bands	356	343	351	354	347		278	274		361	360
MSAP (%)^a^	47.67	45.85	46.37	46.89	46.11		38.60	37.95		49.61	49.74
Fully methylated ratio (%)^b^	46.11	44.43	45.47	45.85	44.95		36.01	35.49		46.76	46.63
Hemi-methylated ratio (%)^c^	1.55	1.42	0.91	1.04	1.17		2.59	2.46		2.85	3.11
	**Toccata**										
	**H_2_O**						**NaCl**			**Recovery**	
**MSAP band type**	**4**	**7**	**14**	**15**	**17**		**7**	**14**		**15**	**17**
I	442	462	472	470	463		409	395		390	389
II	23	20	18	17	19		37	44		25	29
III	149	141	139	140	143		153	168		127	130
IV	169	160	154	156	158		184	176		241	235
Tot. Amplified bands	783	783	783	783	783		783	783		783	783
Tot. methylated bands^a^	341	321	311	313	320		374	388		393	394
Fully methylated bands^b^	318	301	293	296	301		337	344		368	365
MSAP (%)^c^	43.55	40.99	39.71	39.97	40.86		47.76	49.55		50.19	50.31
Fully methylated ratio (%)^d^	40.61	38.44	37.42	37.80	38.44		43.03	43.93		46.99	46.61
Hemi-methylated ratio (%)^e^	2.93	2.55	2.29	2.17	2.42		4.72	5.61		3.19	3.70

^a^(II+III+IV); ^b^(III+IV); ^c^MSAP (%) = [(II+III+IV)/(I+II+III+IV)]x100; ^d^Fully methylated ratio (%) = [(III+IV)/(I+II+III+IV)]x100; ^e^Hemi-methylated bands (%) = [(II)/(I+II+III+IV)]x100. Type I indicated absence of methylation due to the presence of bands in both *Eco*RI/*Hpa*II and *Eco*RI/*Msp*I digest; type II bands appeared only in *Eco*RI/*Hpa*II digestion but not in the *Eco*RI/*Msp*I digest; type III generated bands obtained in *Eco*RI/*Msp*I digest but not in the *Eco*RI/*Hpa*II digest; and type IV represents the absence of band in both enzyme combinations.

### Salinity-induced changes in the level of methylation in genotypes of rapeseed that differ in their tolerance of salinity stress

Consistent with the approach used by Karan et al. [32], all possible banding patterns between control and salinity stress in tolerant and sensitive rapeseed genotypes were compared to identify the changes in cytosine methylation patterns under salinity stress. Sixteen banding patterns were apparent from the MSAP analysis (Table 2, Figure S1). Patterns A–D represent monomorphic classes in which the methylation pattern is the same following either the control or the salinity-stress treatments. Patterns E–J are indicative of cytosine demethylation, whereas possible cytosine methylation events induced by salt stress are represented by patterns K–P [32].

**Table 2:** Analysis of DNA methylation patterns in rapeseed under salinity stress compared with non-stressed conditions.

**Pattern^a^**	**Class**	**H_2_O**			**NaCl**			**Exagone**		**Toccata**
		*Hpa*II	*Msp*I		*Hpa*II	*Msp*I				
**No change**	A	1	0		1	0		4		10
	B	0	1		0	1		133		136
	C	1	1		1	1		366		386
	D	0	0		0	0		117		142
	**Total**							**620 (80.3%)**		**674 (86.1%)**
**Demethylation**	E	1	0		1	1		1		0
	F	0	1		1	1		6		0
	G	0	0		1	1		97		6
	H	0	1		1	0		0		0
	I	0	0		1	0		15		22
	J	0	0		0	1		2		4
	**Total**							**121 (15.7%)**		**32 (4.1%)**
**Methylation**	K	1	1		1	0		2		10
	L	1	1		0	1		1		5
	M	1	1		0	0		23		56
	N	1	0		0	1		0		0
	O	1	0		0	0		2		6
	P	0	1		0	0		3		0
	**Total**							**31 (4%)**		**77 (9.8%)**

^a^ 1 = band present in all stages (4 to 14); 0 = band absent in all stages (4 to 14).

Out of 772 and 783 bands, 80.3% and 86.1% of CCGG sites remained unchanged after the imposition of salinity stress on the tolerant (Exagone) and sensitive (Toccata) cultivars, respectively (Table 2). The percentages of demethylated sites under conditions of salt stress were 15.7% and 4.1% in Exagone and Toccata, respectively (Table 2). This indicated relatively more DNA demethylation events in the salt-tolerant genotype than in the salt-sensitive one (Table 2). The percentages of sites methylated under salt stress were 4% and 9.8% in tolerant and sensitive genotypes, respectively (Table 2). This indicates relatively more DNA methylation events in salt-stressed sensitive than in salt-stressed tolerant genotypes (Table 2). The test of independence between three different methylation patterns and salt treatments, control and salt stress conditions was carried out using chi-square test (Table S2). The chi-square test results suggest association between stress conditions and the level of methylation both in Exagone and Toccata (i.e. 47.66 and 27.37 in Exagone and 58.99 and 30.69 in Toccata at 14 DAS, *P*<0.0001; Table S2).

### Alteration of DNA methylation pattern under salinity stress and after subsequent recovery

To identify the DNA methylation changes (i.e. demethylation or methylation under salinity stress and subsequent recovery), we classified all differentially methylated DNA fragments into various classes. As indicated in Table 3, the **a**, **b**, and **c** classes included bands with DNA demethylation induced by salinity; the **d**, **e**, and **f** classes comprised methylated DNA fragments induced by salinity stress; and the **g** and **h** classes included DNA fragments for which salinity stress had no effect on their methylation status. As many as 71.9% and 84.4% of the demethylated DNA bands were remethylated (class **a**), whereas only 26.4% and 15.6% remained hypomethylated (class **b**) after recovery in Exagone and Toccata, respectively. Only a few bands (2 in Exagone and none in Toccata) were found to belong to class **c** (demethylated by salinity but re-methylated with a different pattern after recovery).

**Table 3:** Summary of the changes in the DNA methylation patterns in Exagone and toccata cultivars after recovery.

Band class*	a	b	c	a+b+c	d	e	f	d+e+f	g	h	i	total
Exagone	87	32	2	**121**	4	21	6	**31**	105	493	22	**772**
Toccata	27	5	0	**32**	28	22	27	**77**	81	575	18	**783**

* (a) demethylated by salinity, but remethylated after recovery; (b) demethylated by salinity, and remaining hypomethylated after recovery; (c) demethylatd by salinity but re-methylatd in a different pattern after recovery; (d) methylated by salinity, but demethylated after recovery; (e) methylated by salinity, and remaining methylated after recovery; (f) methylatd by salinity, but demethylated in a different pattern after recovery; (g) DNA methylation pattern remained unchanged under salinity, but changed after recovery; (h) DNA methylation pattern was unchanged under salinity, and remained unchanged after recovery; (i) others.

Different behaviour was observed in Exagone and Toccata in terms of the methylated DNA fragments induced by salinity stress. For the salt-tolerant Exagone cultivar, recovery affected the methylation status of only a few of the fragments subject to salinity-induced DNA methylation (12.9%, class **d**), whereas the vast majority remained unchanged after recovery from salinity stress (67.8%, class **e**), with only six bands of class **f** (methylated by salinity, but demethylated with a different pattern after recovery). After recovery, the 77 salinity-induced DNA methylated fragments of the salt-sensitive Toccata cultivar were almost equally distributed between the three classes (36.6%, 28.6%, and 35% for classes **d**, **e**, and **f**, respectively).

### Sequencing and bioinformatics analysis of methylated DNA fragments

A total of 19 bands (avg size of 226 bp, ranging from 89 to 363 bp) were excised from acrylamide gels, cloned, and sequenced. Table S3 shows the methylation pattern of all sequenced fragments collected from Exagone and Toccata samples during seedling development under salt stress and control conditions.

Searches for similarities with known genes as well as with genome sequences were undertaken by investigations of the *Brassica* ( *B* *. rapa* and *B. oleracea*) genomes database [45] and the *Arabidopsis* TAIR repository [46] using the BLAST alignment tool [47].

Nine sequences showed at least one significant alignment (e-value lower than 0.05) in either of the two *Brassica* genomes or the *A. thaliana* (Table 4) genome. In particular, five fragments were significantly associated with *B* *. rapa* chromosomes (e-value lower than 0.001), and were also confirmed by alignments with the *B. oleracea* genome sequence. Among these fragments, only Bn_01 overlaps with a coding sequence, which encodes CYP86A8 (Lacerata, LCR), a member of the CYP86A subfamily of cytochrome p450 enzymes (Table 4). In particular Bn_01 is located 181 bp from the start codon. The Bn_02 sequence is located between the Bra027033 (similar to At1g62600, which encodes a flavin-binding monooxygenase protein) and Bra027034 genes (similar to At3g02340, which encodes a RING/U-box superfamily protein) (Table 4 and Table S4). The Bn_03 sequence is located between the Bra034912 (similar to At4g12760, which encodes an unknown protein) and Bra034913 genes (similar to At1g35490 encoding a bZIP family transcription factor) (Table 4 and Table S4). The Bn_05 sequence is located between the Bra023103 (similar to At2g37170, PIP2, Plasma Membrane Intrinsic Protein 2, which is known to be involved in the response to salt stress) and Bra023104 genes (similar to At3g53430, which encodes ribosomal protein L11, involved in RNA methylation) (Table 4 and Table S4). The Bn_06 sequence is located between Bra038073 (similar to At4g15740, which encodes a calcium-dependent lipid-binding protein) and Bra038074 genes (Table 4 and Table S4).

**Table 4:** Genome and functional association of the methylated DNA fragments.

	** *Brassica* *rapa* **		** *B* *. rapa* gene annotation**				** *B* *. Rapa* **†	***B. oleracea***	***Arabidopsis thaliana***			
**ID**	**BLASTn**	**tBLASTx**	**CHR**	**overlap.**	**Gene on**	**Gene on**	**SRA‡**	**BLASTn**	**BLASTn**	**tBLASTx**	**Annotation**	**Gene Ontology**
				**region**	**the left**	**the right**						
**Bn_01**	**	**	V	G	1,96	15,99	**	**	**	**	**AT2G45970** -*LCR*	Fatty acid metabolic process
**Bn_02**	**	**	IX	T	1,14	2,90	**	**		**		
**Bn_03**	**	**	VIII	T	4,68	1,16	**	**		ns		
**Bn_04**	**	**	CHL				**		**	**		
**Bn_05**	**	**	III		0,25	0,58	**	**	*	ns		
**Bn_06**	**	**	VIII		2,57	6,67	**	**	*	ns		
**Bn_07**	ns	**					**	ns	ns	**		
**Bn_08**	ns	**					ns					
**Bn_09**	ns	ns					ns	ns	ns	**	**AT4G27550** - *TPS*4	Trehalose biosynthetic process

** E-value <= 0.001; * 0.001 < E-value <= 0.05; † *B. Rapa*, *B. Rapa* subp. *pekinensis* and *B. rapa* cultivar Chiifu-401-42; ‡Sequence Read Archive at (http://www.ncbi.nlm.nih.gov/sra); **ns**, non-significant alignment; **ΧΗΛ**, chloroplast; **CHR**, Chromosome number; **ID**, MSAP band identification number.

BLAST-based alignments for chromosomal association (BLASTn) and functional annotation (tBLASTx and BLAST2GO) were performed using the *Brassica* *rapa* *and B. oleracea* genomes and *Arabidopsis* genome. Only for fragments that aligned with *B* *. rapa* chromosomes, the overlapping gene (indicated by G) and the transposon element (indicated by T), when present, and the distance (in Kbp) from flanking genes (gene on the left/right) are indicated. The annotations were provided for the overlapping gene in the *B* *. rapa* genome (underlined) or the *A. thaliana* gene (not underlined) using tBLASTx analysis.

Moreover, one fragment was associated with the *B* *. rapa* chloroplast genome (Bn_04) and the last three fragments were only supported by tBLASTx results with either the *Brassica* genomes or the *Arabidopsis* genome. Specifically, Bn_07 and Bn_08 showed similarities to the *B* *. rapa* genome sequence, whereas the Bn_09 sequence overlapped (804 bp from the start codon) with the *A. thaliana* gene, which encodes TPS4 (Trehalose Phosphatase/Synthase 4), a protein involved in trehalose biosynthesis.

### Expression analysis

Expression analysis of the two methylated sequences similar to Lacerata (*LCR*, CYP86A8, Bn_01) and trehalose-6-phosphatase synthase S4 (*TPS*4, Bn_09) was carried out by quantitative RT-PCR on root and shoot samples collected from the two contrasting genotypes under control, salinity stress, and recovery conditions at various seedling developmental stages (from 4 to 17 days after germination, Figure 1). Efficient qRT-PCR amplifications with a single amplicon were achieved for both genes.

**Figure 2:**
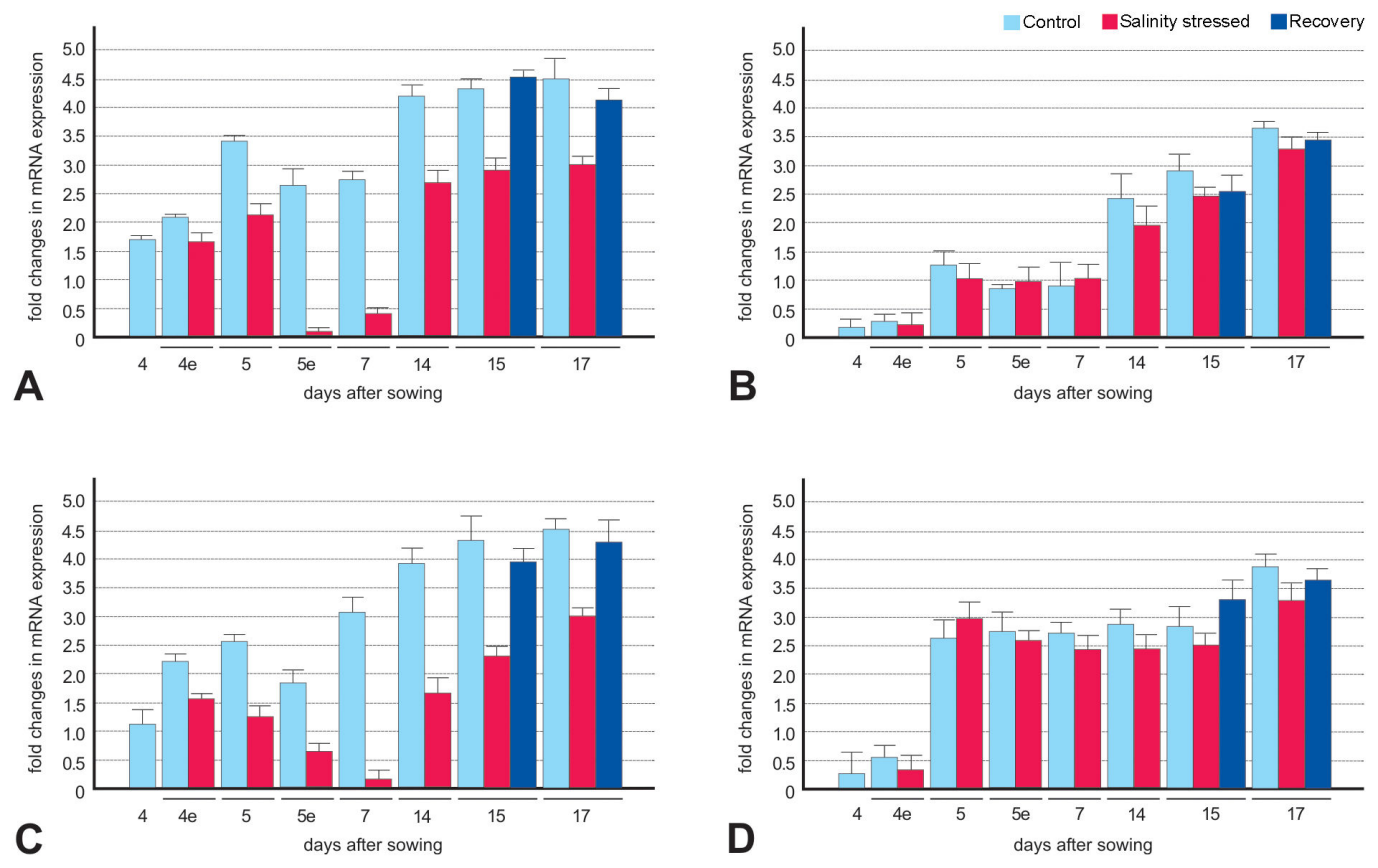
Expression profiles of *Lacerata* (*LCR*) in rapeseed after exposure to salinity stress. Salinity-stressed shoots (A and B) and roots (C and D) of two rapeseed cultivars, Exagone (salt-tolerant, A and C) and Toccata (salt-sensitive, B and D), were used to quantify the expression of *LCR* relative to control. Real-time PCR analysis was performed using gene-specific primers. The expression of *LCR* was normalised relative to the expression of internal control genes, which encode glyceraldehyde-3-phosphate dehydrogenase (*GAPDH*) and elongation factor 1-alpha (*EF-1*- α. Error bars indicate the standard error calculated using a 95% confidence interval. Relative expression values (fold change) are on a logarithm base 2 scale.

Exagone samples grown under non-stressed conditions showed expression of the *LCR* gene at stages 4 and 4e (Figure 2 A and C, light-blue bars) that was much higher than that seen for Toccata (Figure 2 B and D, light-blue bars) in both shoots and roots (P<0.01). Observed differences were statistically significant (Student’s t-test, Table S5). In all other shoot stages, the expression of *LCR* remained higher in Exagone samples, although the differences were smaller (but always statistically significant at at least P<0.01). Not all the differences in LCR expression in the equivalent root stages reached the statistical significance (Table S5). More importantly, *LCR*, which was methylated under salinity stress in the Exagone cultivar, was downregulated after exposure to salinity stress in both shoots and roots of the tolerant Exagone cultivar from stage 4e to stage 17 (Figure 2 A and C, red bars; P<0.01, Table S6), whereas its expression remained unchanged in both shoots and roots of the sensitive Toccata cultivar (Figure 2 B and D, red bars; Table S6). This expression confirms the pattern of methylation and demethylation observed using MSAP (methylated only in Exagone and no methylation changes in Toccata, Table S3). The expression of *LCR* in Exagone samples after recovery from salinity stress rapidly returned to levels comparable to those of samples grown under non-stressed conditions (Figure 2 A and C, blue bars; Table S6).

**Figure 3:**
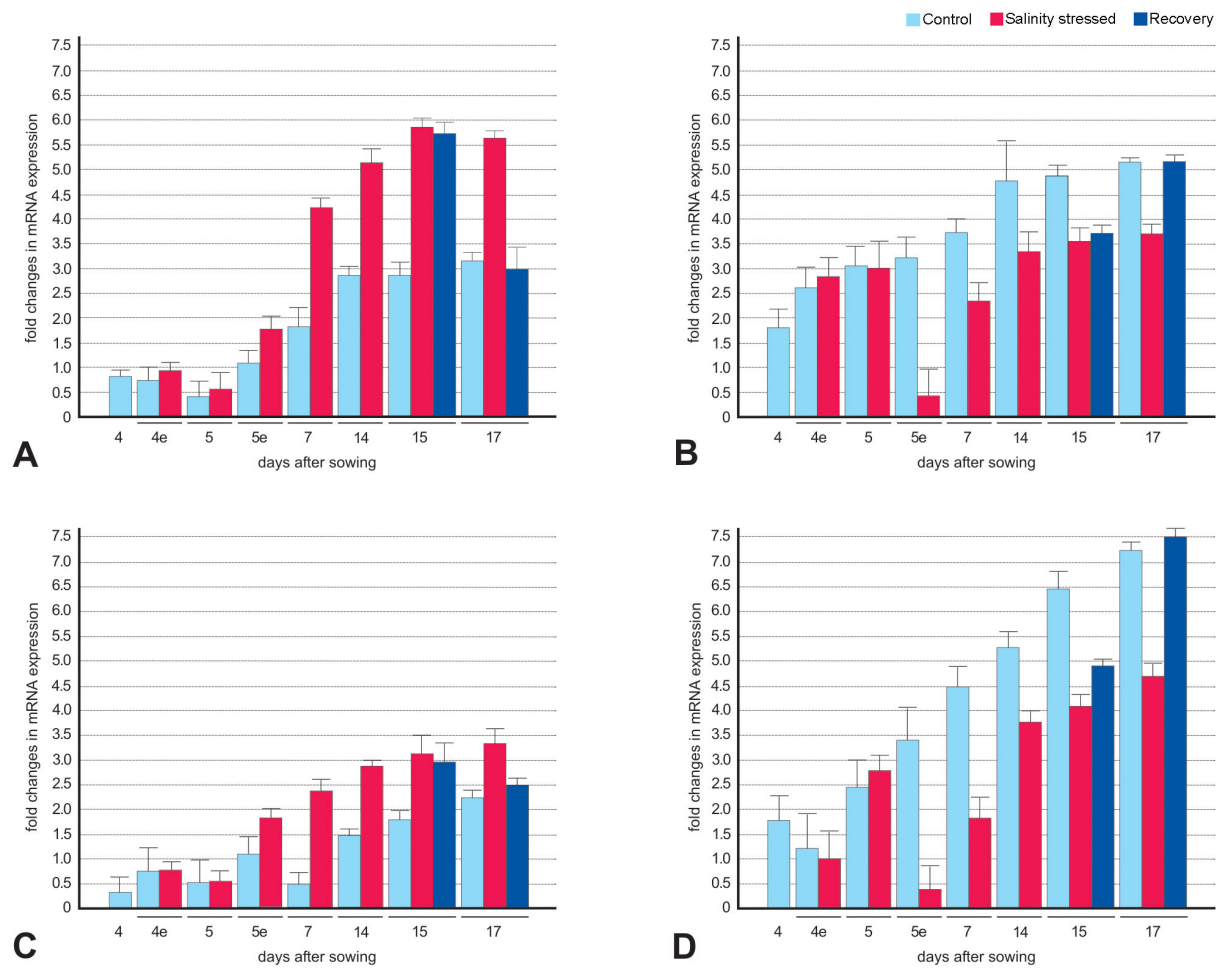
Expression profiles of *TPS4* in rapeseed subjected to salinity stress. Salinity-stressed shoots (A and B) and roots (C and D) of two rapeseed cultivars, Exagone (salt-tolerant) and Toccata (salt-sensitive), were used to quantify levels of *TPS*4 transcripts relative to those in unstressed control plants. Real-time PCR analysis was performed using gene-specific primers. The expression of the *TPS*4 gene was normalised relative to the expression of internal control genes, which encode glyceraldehyde-3-phosphate dehydrogenase (*GAPDH*) and elongation factor 1-alpha (*EF-1*-α. Error bars indicate the standard error calculated using a 95% confidence interval. Relative expression values (fold change) are on a logarithm base 2 scale.

Under control conditions, the overall expression levels of the *TPS*4 gene in Exagone (Figure 3 A and C, light-blue bars) were lower than those for Toccata samples (Figure 3 B and D, light-blue bars) in both shoots and roots at almost all stages (P<0.01, Table S5). Interestingly, in Exagone samples, the *TPS*4 gene was upregulated under salinity stress from stage 5e to stage 17 (Figure 3 A and C, red bars), whereas in Toccata samples, it was downregulated (Figure 3 B and D, red bars) in both shoots and roots compared with the levels in samples grown under control conditions (P<0.01, Table S6). This expression confirms the pattern of methylation and demethylation observed using MSAP (demethylated in Exagone and methylated in Toccata, Table S3). The expression of *TPS*4 in both Exagone and Toccata recovered samples remained at levels comparable to those of samples grown under stressed conditions for a while, and then returned to levels comparable to those of samples grown under non-stressed conditions at stage 17 (Figure 3, blue bars; Table S6).

In order to test the correlation between the expression level of *TPS*4 and the accumulation of trehalose, total soluble sugar was extracted from Exagone leaves and Toccata roots and quantified. We chose these tissues because they were the tissues where the expression of *TPS*4 differed significantly between stressed and control samples. In shoot samples of Exagone the level of trehalose in stressed samples was 2.3 fold that of control samples both at 7 DAS and 14 DAS (Student’s t-test 18.88 and 20.84; P-value 4.6 e-05 and 3.1 e-05, respectively; Table S7). In root samples of Toccata, instead, the level of trehalose did not statistically varied between stressed and control samples (Student’s t-test 0.47 and 0.38; P-value 0.65 and 0.72, respectively; Table S7).

### Bisulfite sequencing

Bisulfite sequencing of *LCR* and *TPS*4 loci was conduct to assess the cytosine methylation status. The bisulfite nucleotide sequences from top strand of these loci were similar to their corresponding loci from *Brassica* ( *B* *. rapa* and *B. oleracea*) genomes database [45] and the *Arabidopsis* TAIR repository [46] and most of the methylated cytosines belonged to CG types (Figures S2 and S3). Alignment of *LCR* (Figure S2) sequence with the bisulfite treated DNA sequences from shoots and roots of Exagone and Toccata under non-stress and salinity stress conditions at 7 and 14 DAS revealed that methylated cytosines present under non-stressing conditions at position 4 and 37 did not change even under salinity stress both in Exagone and Toccata. We found genotype-specific methylations such as the one of cytosine at position 49, which is methylated in both tissues, and both conditions in Exagone but un-methylated in Toccata. Moreover, cytosines at position 145 and 162 are methylated under both conditions in roots of Exagone and Toccata, respectively, but un-methylated in shoots of both cultivars. Finally, and more importantly, cytosine at position 58 (involved in the *Hpa*II/*Msp*I restriction site) is methylated only in Exagone under salt stress.

In case of *TPS*4 (Figure S3) cytosines at 181 and 219 positions were methylated under both conditions and both tissues in Toccata and Exagone, respectively. Cytosine at position 204 was methylated under non-stress and salt stress only in Toccata roots. At position 167 cytosine (belonging to the *Hpa*II/*Msp*I restriction site) was methylated under control conditions in Exagone in both tissues. The methylation status changed in shoots and roots under salt stress. The opposite situation was observed for Toccata. In fact in this genotype, cytosine at position 167 showed to be un-methylated under control conditions and was methylated as consequence of salinity stress.

## Discussion

Plants respond to environmental stresses by adjusting their physiological and developmental machinery through differentially regulated gene expression across the genome [48]. Mechanisms such as DNA methylation and demethylation of cytosine are thought to play a key role in this adjustment [48–51]. Among abiotic constraints limiting plant productivity, salinity is undoubtedly an important and still increasing problem [2,5]. Since most, if not all cultivated plant species originate from salt-sensitive glycophyte species, the selection of improved salt-tolerant cultivars is urgently required. It is now widely accepted that the level of salinity tolerance does not only depend on the presence of specific genes or alleles of specific genes but is also a direct function of the kinetics of gene activation in relation to stress intensity and duration. Stress-induced gene activation has been exhaustively studied through elucidation of transduction pathways and roles of specific transcription factors [52]. Information concerning involvement of methylation/demethylation processes in this respect are still limited and poorly understood, despite their paramount importance for plant breeders.

Under natural field conditions, stresses commonly fluctuate and are rarely permanent. The ability of a cultivated plant species to fully recover after the stress relief is an important component of stress resistance mechanisms [53]. However, to the best of our knowledge, data concerning methylation/demethylation events during recovery phase are rare. The present work considers both a kinetics approach during stress application and a recovery phase after stress relief in relation to DNA methylation process in salt-treated rapeseed.

The MSAP technique [34] was previously applied to study genome methylation in various crops grown under stressed conditions [30-32,37-41]. In this study, the MSAP approach was used to assess whether salt stress caused changes in DNA methylation in salt-tolerant (Exagone) and salt-sensitive (Toccata) rapeseed cultivars [43]. Our results indicate that the level of methylation during seedling development (4 to 17 days after sowing) remains constant in both Exagone (46.57%) and Toccata (41.02%) cultivars. A dramatic change in methylation occurs when plants are grown under salt-stress conditions. However, it is evident that Exagone and Toccata exhibit contrasting behaviour in this regard. The salt-tolerant Exagone cultivar responds to salt stress by decreasing (form an avg of 46.58% to 38.27%) the overall level of DNA methylation, whereas the salt-sensitive Toccata responds by increasing it (form an avg of 41.02% to 48.66%). Moreover, in Exagone the proportion of hemi-methylated bands increased under salinity stress (from an avg of 1.22% to 2.53%), but it remains unclear whether this is due to partial methylation of un-methylated DNA or whether it can be attributed, at least in part, to incomplete demethylation of fully methylated bands.

Using MSAP, Lu et al. [29] assessed DNA modifications in the salt-resistant rapeseed cultivar Westar caused by different saline conditions (from 0 to 200 mmol/L NaCl). In this study, levels of DNA methylation varied from 26.6% (at 0 mmol/L) to 44% (at 200 mmol/L), with a value of 42.6% at 100 mmol/L. When grown under non-stressed conditions (0 mmol/L), both cultivars employed in our study showed a higher level of DNA methylation than that of Westar (46.58% and 41.02% vs. 26.6%). The respective levels of DNA methylation under salinity stress (100 mmol/L) for Exagone and Toccata were lower (38.27%) and higher (48.66%) than those recorded for Westar [29]. Hence, methylation is a direct function of both environment and genotypes.

Besides the total numbers of methylated sequences, it seems that demethylation in Exagone was mainly of the class G, that is, from fully methylated to un-methylated (Table 2). This suggests that in Exagone, genes were activated as a consequence of salinity stress. On the other hand, demethylation in Toccata was mainly of class I, that is, from fully methylated to hemi-methylated (Table 2). For the salt-tolerant Exagone cultivar, our results are consistent with previous observations, i.e. an evident demethylation of genomic DNA caused by environmental factors, such as cold, or excessive levels of heavy metals, aluminium, and salt [27,30,51,54]. Nonetheless, the results in salt-sensitive Toccata seem to contrast with this, showing an increased level of DNA methylation.

Our data also demonstrate a difference in the behaviours of the two cultivars as a result of recovery. The salt-tolerant Exagone mainly showed reversion of demethylated bands, suggesting that physiological and biochemical events occurring during the stress period are reversible. Hence, the plant was able to recover an optimal physiological status after the stress relief. In contrast, for Toccata, the level of methylation at the end of the recovery period was similar to that under stressed conditions. Even if Toccata showed some reversion of both salt-induced methylated and demethylated bands to the original state, our data suggest that Toccata did not fully recover its original status and may be regulating gene expression as if it were still evolving under a “stress context”. It might be argued that Toccata would be able to face an additional stressing events while Exagone would need to activate the whole set of response again. A comparison of our MSAP results with transcriptomic micro-array and proteomic data should afford valuable information to this respect.

The MSAP analysis led us to detect two DNA fragments with sequence similarities to genes that are in some way involved in the response to environmental stresses. The first gene (*LCR*) encodes a cytochrome P450 monooxygenase which catalyses ω-hydroxylation of fatty acids and seems to be involved in the synthesis of cutin and prevention of the accumulation of toxic levels of free fatty acids [55]. In recent years, cuticle-associated proteins have been proven to be very active in plant responses to different stress conditions, and several researchers have suggested that the cuticle plays a significant role in salt stress tolerance in plants [56]. Most *Arabidopsis* mutants show defective cuticles structure and composition. However, there are some defective-cuticle mutants such as *lcr*, which show increased accumulation of cuticle constituents with interesting advantages in response to abiotic stresses [57]. This was also confirmed by Kosma et al [58]. The authors subjected *Arabidopsis* plants to stresses including treatments with sodium chloride (NaCl) and showed a significant increase in cuticular wax. NaCl treatments reduced abundance of LCR transcripts. In another study [59] *lcr* mutants accumulated a level of c18:2 α-ω-diacid three times that of wild type plants and the accumulation of wax was 2 fold that of wild types. Several studies have associated stress induced increases in wax accumulation with major reductions in leaf water-loss rates [58,60]. Our data seem to confirm this hypothesis. In fact, although salt stress does not affect the level of *LCR* transcript in salt-sensitive plants, levels of *LCR* transcript are reduced when salt-tolerant genotypes are subjected to the same stress. This could result in an increase of wax to reduce water loss by plants. Moreover, it seems that Exagone responds efficiently and very rapidly to salt stress by downregulating *LCR* expression in both shoots and roots. In fact, 12 hours after the induction of salt stress (4 to 4e), the level of *LCR* transcripts in our samples was already substantially lower than that observed for plants grown under non-stressed conditions. The rapid response of *LCR* transcript abundance to changes in salinity also occurred during recovery, given that the level of *LCR* transcript at 15 was already comparable to that observed for genotypes grown under non-stress conditions.

The second gene chosen for further characterisation (*TPS*4) acts in the protection against abiotic stress in a large number of organisms, including bacteria, yeast, and invertebrates [61-63]. Even though most species do not seem to accumulate high amounts of trehalose, some studies [64,65] have demonstrated that exogenous application of trehalose significantly reduces damage caused by salt stress in rice. In our samples grown under non-stressed conditions, the level of *TPS*4 transcripts in the salt-sensitive cultivar Toccata was double that in the salt-tolerant cultivar Exagone. Interestingly, under salinity stress, the expression of *TPS*4 increased in the salt-tolerant cultivar and decreased in the salt-sensitive one. This observation is in agreement with the data of Zhang et al. [65], who reported a salt-induced burst of *TPS* expression in the salt-resistant bacteria *Dunaliella* *viridis* . As far as the salt-tolerant cultivar Exagone is concerned, our data demonstrates that salinity indeed significantly increased the endogenous trehalose content in the leaves. Müller et al. [66] recently reported such an increase in response to drought in rapeseed cultivar Titan. Since salt stress clearly presents an osmotic component compromising the plant water status [8,9], the reported increase in response to salt may be at least partly due to salt-induced modification in the plant water potential. In contrast, no stress-induced modification in trehalose content was recorded for the salt-sensitive Toccata. Overall, these data suggest that trehalose could indeed assume positive role in response to salt stress, that *TPS*4 could play a major role in stress-induced increase of trehalose content in salt-resistant cultivars, and that demethylation process might be involved in the gene activation. Responses at recovery were positive, confirming the influence of salinity stress on the expression of *TSP*4. It is worth noting that the response of *TPS*4 to stress in both rapeseed cultivars was slower than that observed for *LCR*. In fact, significant differences in *TPS*4 expression were observed only 36 hours after the induction of salt stress (4-5e), and these differences disappeared only 72 hours after recovery. Although endogenous trehalose may become toxic at high concentrations, it has been reported as an efficient protector of macromolecular structures and may therefore contribute to the stabilization of both proteins and membranes under stressed conditions. Trehalose has also been reported to act as a free radical scavenger, thus helping the plant to reduce the cellular damages induced by stress-induced reactive oxygen species [63,64]. Besides its protective functions, trehalose may also act as an elicitor of genes involved in abiotic stress response [64], and it was shown to confer a high level of salt tolerance in rice, primarily through causing an increase in the synthesis of other soluble sugars [67]. It is noteworthy that Pace et al. [44] recently reported that the level of soluble sugars in rapeseed correlated with tolerance of salinity stress. In particular, they showed that when seeds of the salt-tolerant cultivar Exagone were germinated under conditions of salinity stress, levels of soluble sugars were higher than when Exagone seeds were germinated under non-stressed conditions, as well as those of salt-sensitive Toccata germinated under either stressed and non-stressed conditions. The increase in soluble sugar levels in stressed seedling tissues might thus play an important role in osmotic regulation under salt stress and non-stressed conditions, as is the case for mature plants [68,69].

In conclusion, several genomic studies have revealed that many endogenous genes are methylated either within their promoters or within their transcribed regions, and that gene methylation is highly correlated with transcription levels [19,70,71]. The methylated state is usually associated with inactivation of gene expression and *vice versa* [72]. Moreover, it has also been demonstrated that several genes related to biotic and abiotic stress responses are differentially methylated, which suggests that DNA methylation might be important in plant responses to salt stress. Indeed, we found that salinity could induce genome-wide changes in DNA methylation status, and that these changes, when averaged across different genotypes and developmental stages, accounted for 16.8% of the total site-specific methylation differences in the rapeseed genome, as detected by MSAP analysis. These observations may increase understanding of stress-induced epigenetic impact of stress in plants and to provide more efficient strategies to plant breeders for the selection of resistant cultivars.

## Materials and Methods

### Plant material and growth conditions

Seeds of two rapeseed cultivars, reported to be tolerant (Exagone, Monsanto) and sensitive (Toccata, Maisadour Semences) to salt and drought stress during germination [43], were sterilised in 0.1% NaClO (v/v) and incubated in distilled water (0 mmol/L). Seven samples each of 70 seeds for each cultivar were placed on moistened Whatman No. 1 filter paper in 9-cm-diameter Petri dishes, and incubated under darkness conditions at 20 °C in a controlled chamber. Four days after sowing (i.e. around 1 day after germination), seedlings were transferred to Plexiglas boxes with holes drilled in the lid and walls (21 holes of 3 mm diameter) to perform the slant test [73]. Seedlings were placed 1 cm apart in a horizontal row along the long axis of a 10 × 16 cm rectangular Whatman No. 1 chromatography paper held on a 10 × 18.5 cm clear plastic plate 3 mm thick. The plate was held at an angle of 20° from the vertical by a slotted rack in the base of the 20 × 30 cm seed tray. The paper was kept moist by dipping it into the solution in the tray. Each plate held as many as 10 seedlings, and each tray held 10 plates. Seedlings from each cultivar were placed in boxes containing distilled water (0 mmol/L) or a NaCl solution (100 mmol/L), both added with Flory 9 (Agrimport), a liquid fertiliser for hydroponics. The boxes for the slant test were placed in a growing room for 17 days at day/night temperature of 25/15 °C with a photoperiod of 14 h light (irradiance: 250 µmol m^−2^ s^−1^) and 10 h darkness. A randomised block design with three replicates per treatment (cultivar × growing conditions) was adopted. Stressed and non-stressed plants were retrieved from the plates at specific intervals (Figure 1). After 14 days, some of the samples grown under stress conditions were transferred to non-stressful conditions for 3 days to assess whether exposure of the plants to non-stressful conditions enabled the plants to recover the “normal” patterns of DNA methylation and gene expression (hereafter named “recovered samples”). Samples were collected in triplicate (biological replicates), snap frozen in liquid nitrogen, and stored at -80 °C. Collection was carried out at 8am for samples 4, 5, 7, 14, 15 and 17 (where the number means the days after sowing) and at 8pm for samples 4e, and 5e (Figure 1).

### DNA extraction and methylation sensitive amplified polymorphism (MSAP) analysis

Whole seedling genomic DNA was extracted from triplicate samples collected at 4, 7, 14, 15, and 17 (Figure 1) using the GenElute^TM^ Plant Genomic DNA Miniprep Kit (Sigma-Aldrich). The integrity and size of genomic DNA was checked using 1% agarose gel electrophoresis prior to its use for MSAP analysis. To detect MSAPs, two digestion/ligation reactions were carried out simultaneously. In the first reaction, 500 ng of the genomic DNA was added to a 45 µl mix containing 5 units *Eco*RI, 5 units *Hpa*II (New England Biolabs), 1X Restriction-Ligation buffer (1X One Phor All, Amersham Pharmacia, added with 0.1 M DTT and 250 ng BSA), 50 pmol *Hpa*II adapter, 50 pmol *Eco*RI adapter, 10 mmol ATP, and 1 unit T4 DNA Ligase (Invitrogen). The mixture was incubated at 37 °C for 4 h. The reaction was stopped by incubation at 65 °C for 10 min and then diluted 10 times in 0.1X TE (1 mM Tris–HCl, 0.1 mM EDTA, pH 8). The second digestion/ligation reaction was carried out in the same way, except that *Msp*I was used instead of *Hpa*II.

Two consecutive PCRs were performed to selectively amplify the *Eco*RI-*Hpa*II and *Eco*RI-*Msp*I fragments. The pre-selective amplification was performed using 5 µL of the above-mentioned diluted mixture, which was added to a 15 µL mix containing 50 ng of EcoRI+C or EcoRI+A primer (Table S1), 50 ng of HpaII+T/MspI+T or HpaII+A/MspI+A primer, 1X PCR Buffer (Invitrogen), 5 mM dNTPs (Invitrogen), and 1 unit of Taq DNA polymerase (Invitrogen). The PCR cycling conditions were 1 cycle of 45 s at 94 °C, 30 s at 65°C, and 1 min at 72°C, followed by a touch-down profile (13 cycles with -0.7 °C decrease in temperature for the annealing step in each successive cycle), followed by 18 cycles with annealing at 55.9 °C, and finally by an extension cycle of 10 min at 72 °C.

Selective amplifications of the diluted pre-selective amplified products was carried out using a total of 15 primer combinations (Table S1). For each reaction, 5 µL of 1:10 diluted pre-selective amplified samples was added to the following selective amplification mix: 50 ng of EcoRI+2 or EcoRI+3 primer, 50 ng of HpaII/MspI+3 primer (Table S1), 1X PCR Buffer (Invitrogen), 5 mM of dNTPs (Invitrogen), and 1 unit of Taq DNA polymerase (Invitrogen) in a final volume of 20 µL. The temperature profile for selective amplification PCR reaction was the same as that used for the pre-selection step. One µL of each amplified sample was added to 10 µL of formamide and to 0.5 µL of size standard (Genescan ROX 500, Applied Biosystems). After denaturation (94 °C for 5 min) amplified fragments were separated with the ABI 3130*xl* Genetic Analyzer sequencer.

Amplified fragments were divided into four types based on the presence or absence of bands, which resulted from the differential sensitivity of the fragments to digestion by *Msp*I and *Hpa*II. Type I represents the presence of bands in both enzymes combinations i.e. *Eco*RI/*Hpa*II and *Eco*RI/*Msp*I, type II bands appeared only in *Eco*RI/*Hpa*II but not in the *Eco*RI/*Msp*I, type III generated bands in *Eco*RI/*Msp*I but not in the *Eco*RI/*Hpa*II, and type IV represents the absence of bands following both enzyme combinations. Type II indicates the hemimethylated state of DNA that results from methylation in one DNA strand but not in its complementary strand [33]. Type III represents the case of full CG (internal cytosine) methylation, whereas type IV is the case of full methylation at both cytosines.

### Silver staining and DNA sequences of salt-stress-related fragments

Some samples, which were chosen on the basis of interesting polymorphisms, were run on acrylamide gels and silver stained with the aim of isolating and sequencing the selected bands. To do so, 2 µL of selected samples were added to 1X formamide dye (98% formamide, 10mM EDTA, 0.01% w/v bromophenol blue and 0.01% w/v xylene cyanol) and denatured (heated at 95 °C for 3 min). After denaturation, samples were immediately transferred to iced water for 2 min, loaded onto a 6% denaturing polyacrylamide gel, and run for 3–4 h at 40–55 W at 55 °C. Gels were then silver stained. The gels were fixed by incubation in 10% acetic acid for 20 minutes, washed three times with ultrapure water for 5 min, transferred to a silver impregnation solution (1.5g/L AgNO_3_, 0.056% formaldehyde) for 30 min, and then rinsed 3 times with ultrapure water. Image development was carried out with slow agitation for 6–7 min in developer solution (30g/L Na _2_CO_3_, 0.056% formaldehyde, 400 µg/L sodium thiosulfate). To stop development and to fix the gel, 10% acetic acid was added directly to the developing solution and incubated with shaking for 3 min. The gel was then rinsed briefly in ultrapure water and dried at room temperature.

Interesting polymorphic bands were excised from gels, rehydrated with 100 µL of double-distilled water for 6 h at 4 °C, and stirred frequently. Tubes were centrifuged at 10,000*g* for 10 min, and the supernatant transferred into a fresh tube. Aliquots of 5 µL were used as template for re-amplification by PCR in a 50 µL reaction volume. All PCR reactions were carried out with the same EcoRI+2/3 and Msp/Hpa+3 primer combinations used in selective amplification step with the following profile: 94 °C for 1 min, 30 cycles of denaturation at 94 °C for 1 min, annealing at 55 °C for 1 min, and extension at 72 °C for 1 min, ending with a 10-min extension step at 72 °C.

An aliquot of the re-amplified DNA was cloned into a pCR4-TOPO vector using the TOPO TA cloning kit for sequencing (Invitrogen). Ten plasmid DNAs for each transformation were purified from 5 mL of overnight cultures of *Escherichia coli* in LB medium using the GenElute Plasmid miniprep kit (Sigma). The sequences of both strands of each plasmid were determined after running sequencing reactions (obtained with BigDye^®^ Terminator v3.1 Cycle Sequencing Kit, Applied Biosystems) on an ABI 3130*xl* Genetic Analyzer sequencer (Applied Biosystems).

### Genome-wide mapping of differentially methylated fragments

The sequences were aligned along two *Brassica* genomes, as well as the *Arabidopsis* genome, using different BLAST based approaches [47]. In particular, a BLASTn analysis was used to compare the 24 sequenced bands to the two assembled *Brassica* genomes *B* *. rapa* (Chiifu-401, version 1.1) and *B. oleracea* (version 2011-08-02-BGI), and also for a comparison with the *A. thaliana* (version TAIR10 [46]) genome. A tBLASTx analysis was also performed with the *B* *. rapa* and *A. thaliana* genome sequences in order to identify similarities in protein-coding regions. This type of BLAST translates both the query and the subject sequence in all possible reading frames. In order to confirm the presence of the methylated fragments along the Brassicaceae genomes, we also performed a BLASTn alignment versus The Sequence Read Archive (SRA) [74], where the raw sequence data from *B* *. rapa* and its subspecies *B* *. rapa* *subsp. Pekinensis* (Chinese cabbage) are stored (http://www.ncbi.nlm.nih.gov/Traces/sra/sra.cgi). Finally for each sequence that showed an alignment to the *B* *. rapa* chromosome, the presence of genes both in the alignment region and in the flaking ones were checked, using the Brassica database annotation [45].

In order to enrich the functional description we compiled a Gene Ontology annotation, using the BLAST2GO at the AmiGO web site [75] and setting a cut-off e-value of 0.001.

### RNA isolation and cDNA synthesis

Total RNA was isolated from roots and shoots samples using the GenElute Mammalian Total RNA miniprep Kit (Sigma), following the manufacturer’s instructions. Samples were then treated with RQ1 RNase-free DNase (Promega, Madison, WI) following the manufacturer’s instructions. One µg of total RNA was retro-transcribed using random hexamers with the Superscript III Reverse Transcriptase (Invitrogen) according to the manufacturer’s instructions. The PCR reactions using primers for the two housekeeping genes [76] of *Brassica* *rapa* , GAPDH (Accession no. AF536826; Forward 5’-CCGCTAACTGCCTTGCTCCACTT-3’, Reverse 5’-GCGGCTCTTCCACCTCTCCAGT-3’) and EF-1-α (Accession no. GO479260; Forward 5’-ATACCAGGCTTGAGCATACCG-3’, Reverse 5’-GCCAAAGAGGCCATCAGACAA-3’), were performed on each sample to check the successful of retro-transcription and the absence of DNA contamination.

### Quantitative RT-PCR

All RT-qPCR analyses were performed using an Mx3000P QPCR (Stratagene, La Jolla, CA) system with the SYBR green PCR Master Mix reagent (Applera, Foster City, CA). Using Primer3 software [77] specific primers were designed within the sequences that encode LACERATA (LCR) (Forward 5’-CCGGTACGTATCAGACATGC-3’, Reverse 5’-TCAAAGCGAGTTTTGGGAAT-3’) and trehalose-6-phosphatase synthase S4 (TPS4) (Forward 5’-GCTGATCGTTGGCGTTGA-3’, Reverse 5’-ATGGATTCGCTGCGGAAG-3’). Amplicons of 115 bp and 68 bp were obtained for *LCR* and *TPS4* genes, respectively. The PCR fragments were analysed using a dissociation protocol to ensure that each amplicon was a single product. Amplicons were also sequenced to verify the specificities of the targets. The amplification efficiency was calculated from raw data using the LingRegPCR software [78].

All RT-qPCRs were performed using three biological replicates in a final volume of 25 µl containing 5 µL of cDNA template (previously diluted 1:10), 0.2 µM of each primer, and 12.5 µl of 2× SYBR Green PCR Master Mix (Sigma) according to the manufacturer’s instructions. The following thermal cycling profile was used: 95 °C for 10 min, followed by 50 cycles of 95 °C for 10 s, 57 °C for 15 s, and 72 °C for 15 s. Following cycling, the melting curve was determined in the range 57–95 °C, with a temperature increment of 0.01 °C/sec. Each reaction was run in triplicate (technical replicates). Negative controls included in each run were a reaction conducted in absence of reverse transcriptase and a reaction with no template (2 µL of nuclease-free water instead of 2 µL of cDNA). For negative controls, no signals were observed (data not shown).

Raw Ct data from the MX3000P instrument were exported to a data file and analysed using GeneEx Pro software [79]. During the pre-processing phase, data were corrected for PCR efficiencies and the three technical repeats were averaged. The selected reference genes, *GAPDH* (Accession no. AF536826) and *EF-1-*α (Accession no. GO479260) were subsequently used to normalize Ct values [80-82], and quantities were calculated relative to the maximum Ct value. Because our interest was in fold changes in gene expression between groups, we ultimately converted quantities to a logarithmic scale using a log base 2 conversions, which also allowed us to test the normal distribution of values [83].

### Extraction of total soluble sugars and trehalose quantification

Total soluble sugars were extracted in 80% ethanol. Because of a lack of plant material, sugar analysis was performed in leaves of Exagone and in the roots of Toccata, only. The ethanol fraction was evaporated under vacuum to dryness (Speed-vac) and resuspended in a small fixed volume of HPLC- water (Acros-Organics): 250 µL for the samples derived from the roots and 400 µL for the samples derived from the shoots. Samples were filtered through a 0.2 µm filter (Acrodisc LC13mm-PVDF; Merck-Eurolab). Sugars were separated on an Aminex HPX-87C resin-based column (BIO-RAD: 125-0095) in degassed and prewarmed (70°C) HPLC-water (Acros-Organics) with a flow-rate of 0.6 mL min-1. The column was protected by a precolumn (Micro-guard cartridges, BIO-RAD: 125-0128) and a filter (Filter Services MN724288) and was kept at 80 °C. Detection was performed through a differential refractometer. Standard curves were generated using trehalose obtained commercially (Sigma) and used at five concentrations prepared in HPLC-water (Acros-Organics); the detector response of the standards was linear in this concentration range and the recovery percentages exceeded 93%. Data analysed by the differential refractometer were collected and analysed with the ‘Valuechrom’ chromatography software (BIO-RAD).

### Bisulfite sequencing

Five hundreds nanograms of total DNA extracted from unstressed and salinity stressed shoots and roots of Exagone and Toccata, collected at 7 and 14 DAS, were modified by sodium bisulfite using EZ DNA Methylation-Gold kit (Zymo Research, USA) following the manufacturer’s protocol. An aliquot of 2 µL of bisulfite-treated DNA was used for each PCR reaction (25 µL) using XXX Taq (Life technologies, USA). The primers used for LACERATA locus were: 5’-TTGGTTTAATTGAAAATTGTGAGAGA-3’ (LCR-for) and 5’-TCTCCATCGAAATTAAAAATCCC-3’ (LCR-rev). Primers for TPS4 locus were: 5’-ATTGGGATAGAATTTGAGAGGT-3’ (TPS4-for) and 5’-TTCACTACGAAAATCCCTTT-3’ (TPS4-rev). Amplified products were ligated into the TOPO TA Cloning vector using the TOPO TA Cloning kit for sequencing (Invitrogen). Colonies were grown over night and amplified with specific primer to test the presence of inserts. Plasmids from fifteen positive colonies for each transformation were purified and sequenced. Sequence reactions were performed using Big Dye Terminator v3.1 (Applied Biosystems, Foster City. CA) and run on a ABI 3130xl DNA Analyzer (Applied Biosystems).

Methylation status of DNA was obtained by comparing the sequence of bisulfite-treated DNA with that of untreated DNA, where conversion of a C to T indicated non-methylated C. In contrast, the absence of C to T conversion indicated methylation. Cytosine methylation status in the top strand of obtained nucleotide sequences were calculated using CyMATE v.2 [84]. The methylation level for each of the three kinds of cytosines, CG, CHG, and CHH (where H stands for A, T, or C) was calculated using the following formula: methylated cytosine (%) = [Number of non-converted (methylated) cytosines /Total number of cytosines of each type] x 100.

### Statistical analyses

The chi-square was used to test the independence between methylation level and salt stress condition, using SAS Version 9.2 (SAS Institute, Cary, NC). Student’s t-test was also performed using SAS software.”

## Supporting Information

Figure S1 **Representative MSAP peaks obtained with 7 primer combinations.** Control and salinity-stressed samples were digested with either *Hpa*II or *Msp*I. Arrows and letters indicate the DNA methylation patterns as reported in Table 2. The relative banding patterns are highlighted in blue.
(TIF)

Figure S2 **(**A**) Bisulfite sequencing analysis and (**B**) sequence alignment of sodium bisulfite-modified DNA of *LCR* locus.** Top strand of trehalose was sequenced from the shoots and roots of Exagone and Toccata cultivars under non-stress and salinity stress (100 mmol NaCl) conditions at 7 and 14 Days After Sowing (DAS). Cytosine methylation, CH, CHG, and CHH (H: A, T or C). C: non-stressed; S: Stressed; % mC: percentage of methylated cytosines. Red bars indicate the *Hpa*II/*Msp*I restriction site.
(TIF)

Figure S3 **(**A**) Bisulfite sequencing analysis and (**B**) sequence alignment of sodium bisulfite-modified DNA of *TPS*4 locus.** Top strand of trehalose was sequenced from the shoots and roots of Exagone and Toccata cultivars under non-stress and salinity stress (100 mmol NaCl) conditions at 7 and 14 Days After Sowing (DAS). Cytosine methylation, CH, CHG, and CHH (H: A, T or C). C: non-stressed; S: Stressed; %mC: percentage of methylated cytosines. Red bars indicate the *Hpa*II/*Msp*I restriction site.
(TIF)

Table S1 **MSAP primer combination used.**
(PDF)

Table S2 **Chi-square test for independence calculated to determine whether there was a significant relationship between methylation level and salt stress conditions.**
(PDF)

Table S3 **Methylation pattern of sequenced fragments.** Presence and Absence of bands are represented as 1 and 0, respectively.
(PDF)

Table S4 **Functional classification of methylated fragments.** BLAST based alignments for chromosome association and functional annotation were performed versus both *Brassica* and *Arabidopsis* genomes. For the fragment aligning a *B* *. rapa* chromosome sequence, the annotation of the putative overlapping gene and of the flanking genes (Gene on the left/right) are reported.
(PDF)

Table S5 **Statistical significance of differences in gene expression (salinity-tolerant Exagone vs. salinity-sensitive Toccata) as determined by the Student’s t-test.**
(PDF)

Table S6 **Statistical significance of gene expression differences (non-stress (w) vs. salt-stress (s)/recovery (r)) as determined by the Student’s t-test.**
(PDF)

Table S7 **Trehalose quantification in shoot samples of Exagone and root samples of Toccata.** Analyses were carried out in triplicate. Average values and standard deviations (SD) are reported for each sample. T-Student test was applied to estimate the differences in trehalose accumulation between control and salt-stressed samples at 7 DAS and 14 DAS.
(PDF)
