# Supplementary figures and images for: Use of MSAP Markers to Analyse the Effects of Salt Stress on DNA Methylation in Rapeseed (Brassica napus var. oleifera)
Source: PLoS One. 2013 Sep 23;8(9):e75597. doi: 10.1371/journal.pone.0075597 (PMC3781078; doi:10.1371/journal.pone.0075597)

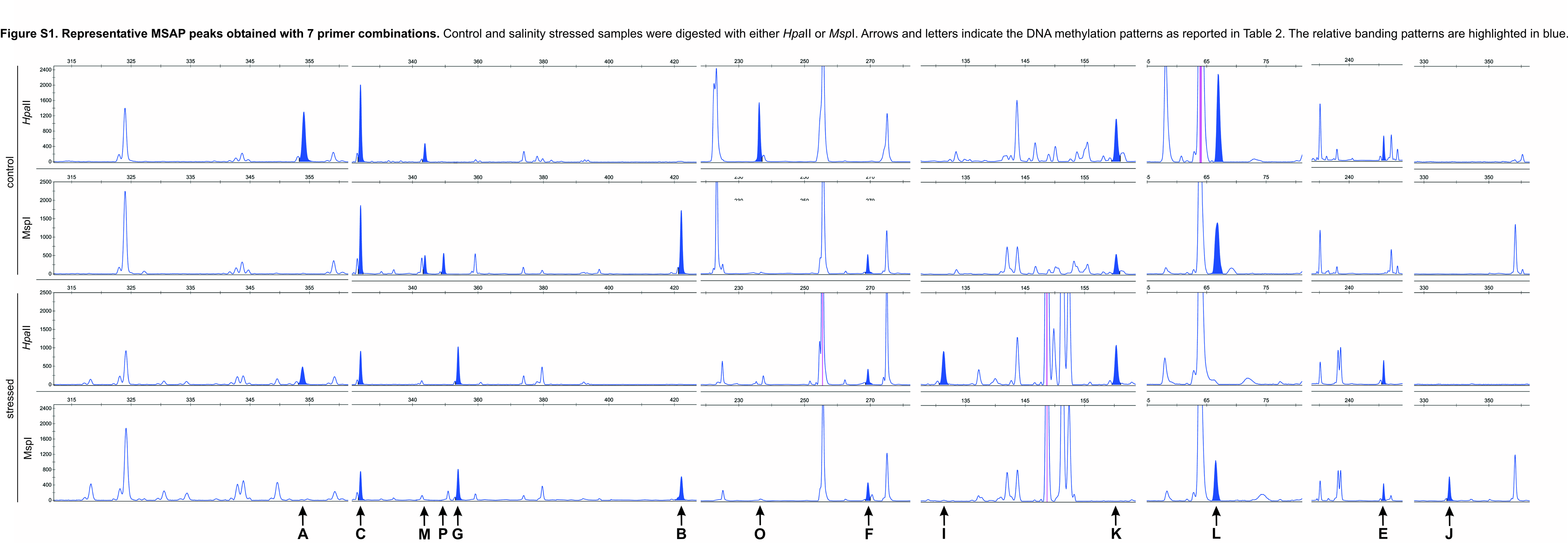

Supplement: Figure S1 — Representative MSAP peaks obtained with 7 primer combinations. Control and salinity-stressed samples were digested with either HpaII or MspI. Arrows and letters indicate the DNA methylation patterns as reported in Table 2. The relative banding patterns are highlighted in blue. (TIF) [file pone.0075597.s001.tif]

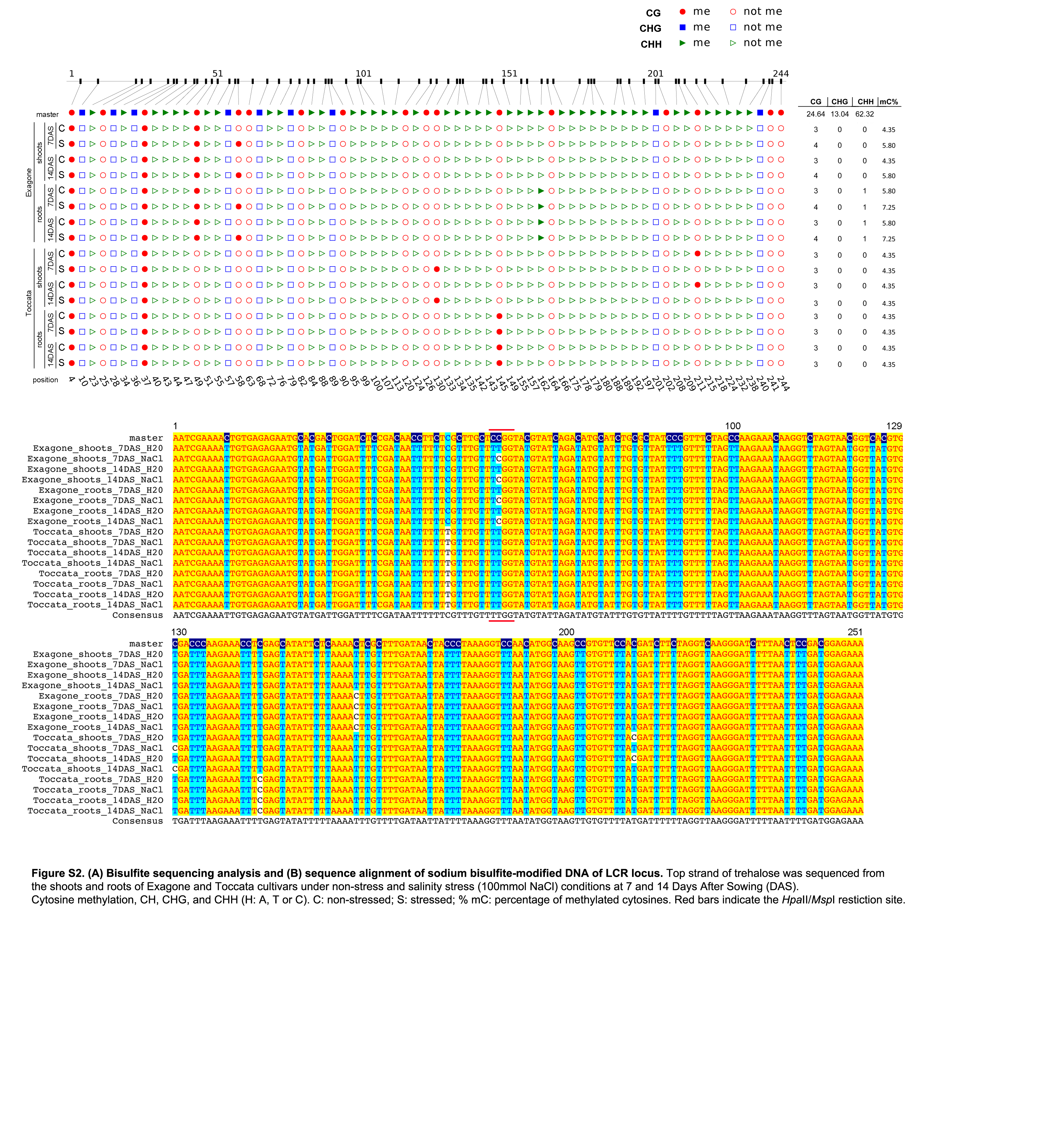

Supplement: Figure S2 — (A) Bisulfite sequencing analysis and (B) sequence alignment of sodium bisulfite-modified DNA of LCR locus. Top strand of trehalose was sequenced from the shoots and roots of Exagone and Toccata cultivars under non-stress and salinity stress (100 mmol NaCl) conditions at 7 and 14 Days After Sowing (DAS). Cytosine methylation, CH, CHG, and CHH (H: A, T or C). C: non-stressed; S: Stressed; % mC: percentage of methylated cytosines. Red bars indicate the HpaII/MspI restriction site. (TIF) [file pone.0075597.s002.tif]

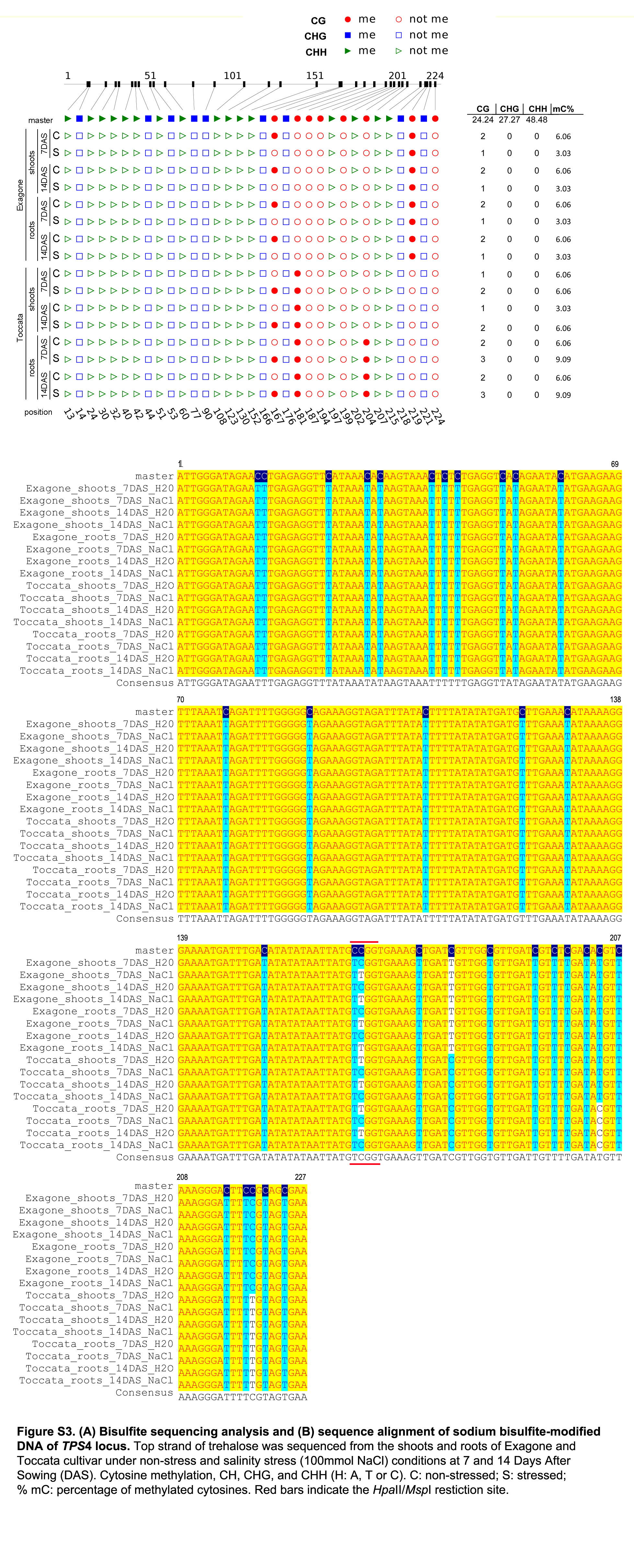

Supplement: Figure S3 — (A) Bisulfite sequencing analysis and (B) sequence alignment of sodium bisulfite-modified DNA of TPS4 locus. Top strand of trehalose was sequenced from the shoots and roots of Exagone and Toccata cultivars under non-stress and salinity stress (100 mmol NaCl) conditions at 7 and 14 Days After Sowing (DAS). Cytosine methylation, CH, CHG, and CHH (H: A, T or C). C: non-stressed; S: Stressed; %mC: percentage of methylated cytosines. Red bars indicate the HpaII/MspI restriction site. (TIF) [file pone.0075597.s003.tif]
