# Supplementary material for: Use of MSAP Markers to Analyse the Effects of Salt Stress on DNA Methylation in Rapeseed (Brassica napus var. oleifera)
Source: PLoS One. 2013 Sep 23;8(9):e75597. doi: 10.1371/journal.pone.0075597 (PMC3781078; doi:10.1371/journal.pone.0075597)
Supplement: Table S1 — MSAP primer combination used. (PDF) [file pone.0075597.s004.pdf]

Table S1. MSAP primer combination used.

Name	Sequence	
ECO RI (E)	5'-GACTGCGTACCAATTC	
HPA/MSP (HM)	5'-GATGAGTCTAGAACGG	
**MSAP primer combinations**		
E-AA/HM-ATC	E-CAA/HM-TGA	E-AGC/HM-ATC
E-CA/HM-TGA	E-CGA/HM-TTA	E-ACT/HM-ATC
E-CC/HM-TGA	E-CCT/HM-TGC	E-AGA/HM-ATC
E-CAA/HM-TAC	E-CAC/HM-TGA	E-AAT/HM-ATT
E-CCC/HM-TCT	E-CCA/HM-TGA	E-AGA/HM-ACT
