# Supplementary material for: Use of MSAP Markers to Analyse the Effects of Salt Stress on DNA Methylation in Rapeseed (Brassica napus var. oleifera)
Source: PLoS One. 2013 Sep 23;8(9):e75597. doi: 10.1371/journal.pone.0075597 (PMC3781078; doi:10.1371/journal.pone.0075597)
Supplement: Table S2 — Chi-square test for independence calculated to determine whether there was a significant relationship between methylation level and salt stress conditions. (PDF) [file pone.0075597.s005.pdf]

Table S2. Chi-square test for independence calculated to determine whether there was a significant relationship between methylation level and salt stress conditions.

**Exagone**							
**Treatment**	**MSAP band type**	**Number of loci (7 days)**	**Number of loci (14 days)**	**Treatment**	**MSAP band type**	**Number of loci (7 days)**	**Number of loci (14 days)**
H_2O	Un-methylated	418	414	H_2O	Un-methylated	410	416
H_2O	Hemi-methylated	11	7	H_2O	Hemi-methylated	8	9
H_2O	Fully-methylated	343	351	H_2O	Fully-methylated	354	347
NaCl	Un-methylated	474	479	Recovery	Un-methylated	389	388
NaCl	Hemi-methylated	20	19	Recovery	Hemi-methylated	22	24
NaCl	Fully-methylated	278	274	Recovery	Fully-methylated	361	360
	**Chi-square test**	27.184	47.668		**Chi-square test**	25.714	27.372
	P-value	<0.0001	<0.0001		P-value	<0.0001	<0.0001
	DF	2	2		DF	2	2

Toccata							
**Treatment**	**MSAP band type**	**Number of loci (7 days)**	**Number of loci (14 days)**	**Treatment**	**MSAP band type**	**Number of loci (7 days)**	**Number of loci (14 days)**
H_2O	Un-methylated	462	472	H_2O	Un-methylated	470	463
H_2O	Hemi-methylated	20	18	H_2O	Hemi-methylated	17	19
H_2O	Fully-methylated	301	293	H_2O	Fully-methylated	296	301
NaCl	Un-methylated	409	395	Recovery	Un-methylated	390	389
NaCl	Hemi-methylated	37	44	Recovery	Hemi-methylated	25	29
NaCl	Fully-methylated	337	344	Recovery	Fully-methylated	368	365
	**Chi-square test**	24.836	58.994		**Chi-square test**	34.895	30.698
	P-value	<0.0001	<0.0001		P-value	<0.0001	<0.0001
	DF	2	2		DF	2	2
