# Supplementary material for: Use of MSAP Markers to Analyse the Effects of Salt Stress on DNA Methylation in Rapeseed (Brassica napus var. oleifera)
Source: PLoS One. 2013 Sep 23;8(9):e75597. doi: 10.1371/journal.pone.0075597 (PMC3781078; doi:10.1371/journal.pone.0075597)
Supplement: Table S3 — Methylation pattern of sequenced fragments. Presence and Absence of bands are represented as 1 and 0, respectively. (PDF) [file pone.0075597.s006.pdf]

Table S3. Methylation pattern of sequenced fragments. Presence and Absence of bands are represented as 1 and 0, respectively.

		Exagone																		Toccata																			
		*Msp*I									*Hpa*II									*Msp*I									*Hpa*II										
		H20					NaCl		Recovery		H20					NaCl		Recovery		H20					NaCl		Recovery		H20					NaCl		Recovery	
**IDs**	**length (bp)**	**4**	**7**	**14**	**15**	**17**	**7**	**14**	**15**	**17**	**4**	**7**	**14**	**15**	**17**	**7**	**14**	**15**	**17**	**4**	**7**	**14**	**15**	**17**	**7**	**14**	**15**	**17**	**4**	**7**	**14**	**15**	**17**	**7**	**14**	**15**	**17**
Bn_01	233	1	1	1	1	1	0	0	1	1	1	1	1	1	1	1	1	1	1	0	0	0	0	0	0	0	0	0	0	0	0	0	0	0	0	0	0
Bn_02	363	0	0	0	0	0	1	1	0	0	0	0	0	0	0	1	1	0	0	0	0	0	0	0	0	0	0	0	0	0	0	0	0	0	0	0	0
Bn_03	300	1	1	1	1	1	0	0	0	0	1	1	1	1	1	1	1	1	1	1	1	1	1	1	1	1	1	1	1	1	1	1	1	1	1	1	1
Bn_04	168	0	0	0	0	0	0	0	1	1	0	0	0	0	0	0	0	1	1	0	1	1	1	1	0	0	1	1	0	1	1	1	1	0	0	1	1
Bn_05	120	0	0	0	0	0	1	1	1	1	0	0	0	0	0	1	1	1	1	0	0	0	0	0	0	0	0	0	0	0	0	0	0	0	0	0	0
Bn_06	113	1	1	1	1	1	0	0	0	0	0	0	0	0	0	0	0	0	0	0	0	0	0	0	1	1	1	1	0	0	0	0	0	0	0	0	0
Bn_07	266	0	0	0	0	0	0	0	0	0	0	0	0	0	0	1	1	0	0	0	0	0	0	0	0	0	0	0	0	0	0	0	0	0	0	0	0
Bn_08	325	1	1	1	1	1	1	1	1	1	1	1	1	1	1	1	1	0	0	1	1	1	1	1	1	1	1	1	0	0	0	0	0	0	0	0	0
Bn_09	99	0	0	0	0	0	1	1	0	0	0	0	0	0	0	1	1	0	0	1	1	1	1	1	0	0	1	1	1	1	1	1	1	0	0	1	1
Bn_10	170	0	0	0	0	0	0	0	0	0	0	0	0	0	0	0	0	0	0	1	1	1	1	1	0	0	0	0	1	1	1	1	1	0	0	0	0
Bn_11	119	0	0	0	0	0	0	0	0	0	0	0	0	0	0	0	0	0	0	0	0	0	0	0	1	1	1	1	1	1	1	1	1	0	0	0	0
Bn_12	89	0	0	0	0	0	1	1	0	0	0	0	0	0	0	0	0	0	0	0	0	0	0	0	0	0	0	0	0	0	0	0	0	0	0	0	0
Bn_13	151	1	1	1	1	1	0	0	0	0	1	1	1	1	1	0	0	0	0	0	0	0	0	0	0	0	0	0	0	0	0	0	0	1	1	0	0
Bn_14	150	0	0	0	0	0	1	1	0	0	0	0	0	0	0	0	0	0	0	0	0	0	0	0	0	0	0	0	0	0	0	0	0	0	0	0	0
Bn_15	144	1	1	0	0	0	0	0	0	0	1	1	0	0	0	0	0	0	0	0	0	0	0	0	0	0	0	0	0	0	0	0	0	0	0	0	0
Bn_16	217	0	0	0	0	0	1	1	0	0	1	1	1	1	1	1	1	1	1	0	0	0	0	0	1	0	0	0	1	1	1	1	1	1	1	1	1
Bn_17	187	1	1	1	1	1	0	0	0	0	1	1	1	1	1	0	0	0	0	0	0	0	0	0	1	0	0	0	0	0	0	0	0	1	1	0	0
Bn_18	156	1	1	1	1	1	0	0	0	0	0	0	0	0	0	0	0	0	0	0	0	0	0	0	0	1	1	1	0	0	0	0	0	0	0	0	0
Bn_19	144	0	0	0	0	0	1	1	0	0	0	0	0	0	0	1	1	0	0	1	1	1	1	1	0	0	0	0	1	1	1	1	1	0	0	0	0
