# Supplementary material for: Use of MSAP Markers to Analyse the Effects of Salt Stress on DNA Methylation in Rapeseed (Brassica napus var. oleifera)
Source: PLoS One. 2013 Sep 23;8(9):e75597. doi: 10.1371/journal.pone.0075597 (PMC3781078; doi:10.1371/journal.pone.0075597)
Supplement: Table S4 — Functional classification of methylated fragments. BLAST based alignments for chromosome association and functional annotation were performed versus both Brassica and Arabidopsis genomes. For the fragment aligning a B . rapa chromosome sequence, the annotation of the putative overlapping gene and of the flanking genes (Gene on the left/right) are reported. (PDF) [file pone.0075597.s007.pdf]

Table S4. Funcional classification of methylated fragments. BLAST based alignments for chromosome association and functional annotation were performed versus both *Brassica* and *Arabidopsis* genomes. For the fragment aligning a B. rapa chromosome sequence, the annotation of the putative overlapping gene and of the flanking genes (Gene on the left/right) are reported.

Brassica rapa

		BLASTn				tBLASTx				Overlapping gene		
IDs	length	chromosome	score	evalue	genomic region	chromosome	score	evalue	genomic region	Gene ID	Gene Annotation	Transposon
Bn_01	134	V	359	4E-98	2619297 - 2619525	V	194	1E-49	2619523 - 2619293	Bra004951	similar to AT2G45970 - fatty acid (omega-1)-hydroxylase/oxygen binding GO:0006631:fatty acid metabolic	
Bn_02	363	IX	507	1E-142	7773082 - 7773447	IX	138	2E-62	7773445 - 7773272			yes
Bn_03	300	VIII	186	5E-46	5337782 - 5337513	VIII	157	3E-38	5337483 - 5337782			yes
Bn_04	168	chloroplast	296	1E-46	75643 - 75543	chloroplast	517	3E-18	75380 - 75280			
Bn_05	120	III	208	5E-53	8707996 - 8707876	III	68	3E-16	8707878 - 8707961			
Bn_06	113	VIII	192	2E-48	6744377 - 6744281	VIII	82	3E-16	6744281 - 6744382			
Bn_07	266					III	47	1E-08	19385783 - 19385691			
Bn_08	325					II	47	0,00006	21750725 - 21750955			
Bn_09	99											

	Gene on the left				Gene on the right			
IDs	dist(Kb)	strand	Gene ID	Gene Annotation	dist(Kb)	strand	Gene ID	Gene Annotation
Bn_01	1,96	+	Bra004950	similar to AT2G45960 | water channel	15,99	-	Bra004952	similar to AT2G45980 - unknown protein
Bn_02	1,14	-	Bra027033	similar to AT1G62600 - flavin-containing monooxygenase family protein	2,90	+	Bra027034	similar to AT3G02340 - zinc finger (C3HC4-type RING finger) family protein
Bn_03	4,68	-	Bra034913	similar to AT1G35490 - bZIP family transcription factor	1,16	+	Bra034912	similar to AT4G12760 - unknown protein
Bn_04								
Bn_05	0,25	+	Bra023103	similar to AT2G37170 - water channel	0,58	-	Bra023104	similar to AT3G53430 - 60S ribosomal protein L12 (RPL12B)
Bn_06	2,57	+	Bra038074	n/a	6,67	-	Bra038073	similar to AT4G15740 - C2 domain-containing protein
Bn_07								
Bn_08								
Bn_09								

	Brassica oleracea			Sequence Read Archive (SRA)					
	blastn			*B. Rapa*		*B. Rapa subp. pekinensis*		*B. rapa* cultivar Chiifu-401-42	
IDs	chromosome	score	evalue	query coverage (max)	evalue (max)	query coverage (max)	evalue (max)	query coverage (max)	evalue (max)
Bn_01	IV	454	1,00E-126	99%	1E-51	99%	2E-53	100%	4E-99
Bn_02	VI	720	0	33%	6,E-05	12%	0,69	100%	8E-152
Bn_03		595	1,00E-168	28%	4,E-08			100%	3E-81
Bn_04				60%	2E-43	60%	9E-44	56%	2E-14
Bn_05	III	167	3,E-40	32%	1,4			100%	7E-51
Bn_06	VIII	131	1,E-29	85%	6E-43	15%	8,3	85%	3E-43
Bn_07	IV	38	0,42			83%	5E-53	8%	2,2
Bn_08								16%	2,2
Bn_09	VII	34	2,1			18%	2,4	26%	0,64

	Arabidopsis thaliana						
	BLASTn			tBLASTx			blast2GO
IDs	chromosome	score	evalue	Gene_ID | Annotation	score	evalue	GeneOntology
Bn_01	II	297	5,00E-80	AT2G45970 - CYP86A8, Lacerata (LCR)	103	3,E-23	**GO:0006631** (fatty acid metabolic process)
Bn_02				AT4G22505 - Bifunctional inhibitor/lipid-transfer	41	5,E-07	**GO:0006869** (lipid transport)
Bn_03				AT3G18810 - Protein kinase superfamily	35	7,E-02	
Bn_04	chloroplast	168	2,00E-41	ATCG00730 - Photosynthetic electron transfer D (chloroplast)	58	3,E-09	**GO:0006355** (regulation of transcription, DNA-dependent)
Bn_05	V	41	0,007				
Bn_06	II	39,2	0,023				
Bn_07	I	37,4	0,081	AT3G24090 - Glutamine-fructose-6-phosphate transaminase	47	7,E-09	**GO:0006040** (amino sugar metabolic process)
Bn_08							
Bn_09	IV	35,6	0,28	AT4G27550 - Trehalose-6-phosphatase/synthetase 4(TPS4)	35	1,E-04	**GO:0005992** (trehalose biosynthetic process)
