# Supplementary material for: Use of MSAP Markers to Analyse the Effects of Salt Stress on DNA Methylation in Rapeseed (Brassica napus var. oleifera)
Source: PLoS One. 2013 Sep 23;8(9):e75597. doi: 10.1371/journal.pone.0075597 (PMC3781078; doi:10.1371/journal.pone.0075597)
Supplement: Table S5 — Statistical significance of differences in gene expression (salinity-tolerant Exagone vs. salinity-sensitive Toccata) as determined by the Student’s t-test. (PDF) [file pone.0075597.s008.pdf]

Table S5. Statistical significance of differences in gene expression (salinity-tolerant Exagone vs. salinity-sensitive Toccata) as determined by the Student's *t*-test.

LCR gene																							
shoot/water Exagone vs Toccata						root/water Exagone vs Toccata						shoot/salt Exagone vs Toccata						root/salt tolerant vs sensitive					
**DAS**	**avg**	**SD**	**avg**	**SD**	**P-value**	**DAS**	**avg**	**SD**	**avg**	**SD**	**P-value**	**DAS**	**avg**	**SD**	**avg**	**SD**	**P-value**	**DAS**	**avg**	**SD**	**avg**	**SD**	**P-value**
**4**	1,6967	0,0611	0,1567	0,1266	0,0004	**4**	1,1100	0,2663	0,2600	0,3751	0,0381	**4**						**4**					
**4e**	2,0667	0,0603	0,2567	0,1002	5,87507E-05	**4e**	2,1900	0,1513	0,5400	0,2179	0,0008	**4e**	1,6700	0,1300	0,1933	0,1701	0,0004	**4e**	1,5433	0,0839	0,3400	0,2587	0,0095
**5**	3,4100	0,0954	1,2467	0,2139	0,0008	**5**	2,5367	0,1242	2,6233	0,3075	0,6854	**5**	2,1133	0,2026	1,0100	0,3035	0,0092	**5**	1,2500	0,1992	2,9800	0,2972	0,0020
**5e**	2,6367	0,2831	0,8333	0,0702	0,0057	**5e**	1,8167	0,2178	2,7500	0,3568	0,0256	**5e**	0,0767	0,0666	0,9667	0,2495	0,0195	**5e**	0,6500	0,1480	2,5833	0,1893	0,0002
**7**	2,7300	0,1510	0,8933	0,4131	0,0095	**7**	3,0500	0,2706	2,7100	0,1873	0,1569	**7**	0,3833	0,1242	1,0167	0,2757	0,0409	**7**	0,1633	0,1484	2,4400	0,2536	0,0006
**14**	4,1867	0,2155	2,4300	0,4293	0,0084	**14**	3,8967	0,2631	2,8733	0,2658	0,0090	**14**	2,5700	0,2427	1,9533	0,3544	0,0517	**14**	1,6600	0,2458	2,4467	0,2409	0,0167
**15**	4,3267	0,1563	2,9167	0,2994	0,0054	**15**	4,2867	0,4366	2,9400	0,2800	0,0156	**15**	2,6700	0,2427	2,5533	0,2873	0,6195	**15**	2,3567	0,1150	2,5000	0,2066	0,0693
**17**	4,5133	0,3287	3,6533	0,0802	0,0389	**17**	4,4867	0,1861	3,8700	0,2685	0,0365	**17**	3,1000	0,1997	3,1833	0,1890	0,6019	**17**	3,0500	0,2706	3,3800	0,1836	0,0519

TPS4 gene																							
shoot/water Exagone vs Toccata						root/water Exagone vs Toccata						shoot/salt Exagone vs Toccata						root/salt tolerant vs sensitive					
**DAS**	**avg**	**SD**	**avg**	**SD**	**P-value**	**DAS**	**avg**	**SD**	**avg**	**SD**	**P-value**	**DAS**	**avg**	**SD**	**avg**	**SD**	**P-value**	**DAS**	**avg**	**SD**	**avg**	**SD**	**P-value**
**4**	0,8433	0,1124	1,8067	0,3408	0,0294	**4**	0,3233	0,2916	1,7833	0,5132	0,0209	**4**						**4**					
**4e**	0,7467	0,2548	2,5933	0,3921	0,0041	**4e**	0,7467	0,4772	1,2467	0,6885	0,3663	**4e**	0,9100	0,1709	2,8100	0,3676	0,0048	**4e**	0,7800	0,1652	1,0200	0,5631	0,5426
**5**	0,3733	0,3288	2,9967	0,4010	0,0011	**5**	0,5033	0,4626	2,4667	0,5534	0,0099	**5**	0,5367	0,3400	2,9833	0,5154	0,0039	**5**	0,5433	0,2031	2,7867	0,3301	0,0013
**5e**	1,0833	0,2413	3,1467	0,4501	0,0056	**5e**	1,0700	0,3747	3,4267	0,6634	0,0111	**5e**	1,7467	0,2650	0,4200	0,5260	0,0307	**5e**	1,8200	0,1905	0,3867	0,3711	0,0096
**7**	1,8000	0,4015	3,6867	0,2554	0,0042	**7**	0,4667	0,2335	4,4967	0,4274	0,0006	**7**	4,1800	0,1970	2,3133	0,3668	0,0041	**7**	2,3767	0,2363	1,8300	0,4321	0,1473
**14**	2,8233	0,1793	4,6933	0,8050	0,0506	**14**	1,4600	0,1353	5,3100	0,3635	0,0011	**14**	5,0767	0,2650	3,2867	0,3819	0,0039	**14**	2,8833	0,1159	3,8033	0,2307	0,0090
**15**	2,8333	0,2608	4,7933	0,2113	0,0007	**15**	1,7867	0,1747	6,4633	0,3584	0,0003	**15**	5,6600	0,2095	3,5200	0,2689	0,0003	**15**	3,0800	0,4107	4,1367	0,3510	0,0468
**17**	3,1200	0,1825	5,0567	0,3024	0,0017	**17**	2,2467	0,1595	7,2533	0,1861	4,7854E-06	**17**	5,6267	0,0569	3,6200	0,1931	0,0002	**17**	3,3133	0,4528	4,7600	0,2402	0,0019
