# Supplementary material for: Use of MSAP Markers to Analyse the Effects of Salt Stress on DNA Methylation in Rapeseed (Brassica napus var. oleifera)
Source: PLoS One. 2013 Sep 23;8(9):e75597. doi: 10.1371/journal.pone.0075597 (PMC3781078; doi:10.1371/journal.pone.0075597)
Supplement: Table S6 — Statistical significance of gene expression differences (non-stress (w) vs. salt-stress (s)/recovery (r)) as determined by the Student’s t-test. (PDF) [file pone.0075597.s009.pdf]

Table S6. Statistical significance of gene expression differences (non-stress (w) vs. salt-stress (s)/recovery (r)) as determined by the Student's *t*-test.

LCR gene

Exagone

shoots

DAS	avg (w)	SD (w)	avg (s)	SD (s)	avg (r)	SD (r)	P-value
**4**							
**4e**	2,0667	0,0603	1,6700	0,1300			0,00991
**5**	3,4100	0,0954	2,1133	0,2026			0,00132
**5e**	2,6367	0,2831	0,0767	0,0666			0,00136
**7**	2,7300	0,1510	0,3833	0,1242			0,00002
**14**	4,1867	0,2155	2,5700	0,2427			0,00067
**15**	4,3267	0,1563	2,6700	0,2427			0,00058
**15**	4,3267	0,1563			48,5800	0,17435596	0,13423
**15**			2,6700	0,2427	48,5800	0,17435596	0,00038
**17**	4,5233	0,1563	3,1000	0,1997			0,00063
**17**	4,5233	0,1563			4,0766	0,3828	0,13467
**17**			3,1000	0,1997	4,0766	0,3828	0,01728

roots

DAS	avg (w)	SD (w)	avg (s)	SD (s)	avg (r)	SD (r)	P-value
4							
4e	2,1900	0,1513	1,5433	0,0839			0,0033
5	2,5367	0,1242	1,2500	0,1992			0,0008
5e	1,8167	0,2178	0,6500	0,1480			0,0013
7	3,0500	0,2706	0,1633	0,1484			0,0002
14	3,8967	0,2631	1,6600	0,2458			0,0002
15	4,2867	0,4366	2,3567	0,1150			0,0018
15	4,2867	0,4366			3,8800	0,2443	0,2320
15			2,3567	0,1150	3,8800	0,2443	0,0006
17	4,5333	0,3881	3,0500	0,2706			0,0056
17	4,5333	0,3881			4,3500	0,1442	0,4859
17			3,0500	0,2706	4,3500	0,1442	0,0018

Toccata

shoots

DAS	avg (w)	SD (w)	avg (s)	SD (s)	avg (r)	SD (r)	P-value
**4**							
**4e**	0,2567	0,1002	0,1933	0,1701			0,3073
**5**	1,2467	0,2139	1,0100	0,3035			0,1690
**5e**	0,8333	0,0702	0,9667	0,2495			0,2278
**7**	0,8933	0,4131	1,0167	0,2757			0,3462
**14**	2,4300	0,4293	1,9533	0,3544			0,1073
**15**	2,9167	0,2994	2,5533	0,2873			0,2039
**15**	2,9167	0,2994			2,6133	0,2977	0,2813
**15**			2,5533	0,2873	2,6133	0,2977	0,8140
**17**	3,6533	0,1802	3,1833	0,1890			0,0566
**17**	3,6533	0,0802			3,4666	0,3958	0,4682
**17**			3,1833	0,1890	3,4666	0,3958	0,3258

roots

DAS	avg (w)	SD (w)	avg (s)	SD (s)	avg (r)	SD (r)	P-value
**4**							
**4e**	0,5400	0,2179	0,3400	0,2587			0,1826
**5**	2,6233	0,3075	2,9800	0,2972			0,1111
**5e**	2,7500	0,3568	2,5833	0,1893			0,2629
**7**	2,7100	0,1873	2,4400	0,2536			0,1090
**14**	2,8733	0,2658	2,4467	0,2409			0,0545
**15**	2,9400	0,2800	2,5000	0,2066			0,0937
**15**	2,9400	0,2800			3,3633	0,2558	0,1253
**15**			2,5000	0,2066	3,3633	0,2558	0,0104
**17**	3,7867	0,2153	3,3800	0,2836			0,0630
**17**	3,7867	0,1153			3,5833	0,1893	0,1992
**17**			3,3800	0,1836	3,5833	0,1893	0,1992

*TPS*4 gene

Exagone

shoots

DAS	avg (w)	SD (w)	avg (s)	SD (s)	avg (r)	SD (r)	P-value
**4**							
**4e**	0,7467	0,2548	0,9100	0,1709			0,4156
**5**	0,3733	0,3288	0,5367	0,3400			0,5821
**5e**	1,0833	0,2413	1,7467	0,2650			0,0331
**7**	1,8000	0,4015	4,1800	0,1970			0,0030
**14**	2,8233	0,1793	5,0767	0,2650			0,0005
**15**	2,8333	0,2608	5,6600	0,2095			0,0001
**15**	2,8333	0,2608			5,7833	0,1595	0,0001
**15**			5,6600	0,2095	5,7833	0,1595	0,4627
**17**	3,1200	0,1825	5,6267	0,0569			0,00002
**17**	3,1200	0,1825			3,0466	0,4980	0,8225
**17**			5,6267	0,0569	3,0466	0,4980	0,0009

roots

DAS	avg (w)	SD (w)	avg (s)	SD (s)	avg (r)	SD (r)	P-value
**4**							
**4e**	0,7467	0,4772	0,7800	0,1652			0,9176
**5**	0,5033	0,4626	0,5433	0,2031			0,9004
**5e**	1,0700	0,3747	1,8200	0,1905			0,0544
**7**	0,4667	0,2335	2,3767	0,2363			0,0006
**14**	1,4600	0,1353	2,8833	0,1159			0,0002
**15**	1,7867	0,1747	3,1766	0,2250			0,0011
**15**	1,7867	0,1747			2,9800	0,4458	0,0125
**15**			3,1766	0,2250	2,9800	0,4458	0,5326
**17**	2,2467	0,1595	3,3866	0,1106			0,0005
**17**	2,2467	0,1595			2,5066	0,2053	0,1582
**17**			3,3866	0,1106	2,5066	0,2053	0,0028

Toccata

shoots

DAS	avg (w)	SD (w)	avg (s)	SD (s)	avg (r)	SD (r)	P-value
**4**							
**4e**	2,5933	0,3921	2,8100	0,3676			0,5236
**5**	2,9967	0,4010	2,9833	0,5154			0,9736
**5e**	3,1467	0,4501	0,4200	0,5260			0,0026
**7**	3,6867	0,2554	2,3133	0,3668			0,0082
**14**	4,6933	0,8050	3,2867	0,3819			0,0457
**15**	4,7933	0,2113	3,5200	0,2689			0,0030
**15**	4,7933	0,2113			3,6100	0,2706	0,0040
**15**			3,5200	0,2689	3,6100	0,2706	0,7037
**17**	5,0567	0,3024	3,6200	0,1931			0,0023
**17**	5,0567	0,3024			5,0700	0,3245	0,9610
**17**			3,6200	0,1931	5,0700	0,3245	0,0027

roots

DAS	avg (w)	SD (w)	avg (s)	SD (s)	avg (r)	SD (r)	P-value
**4**							
**4e**	1,2467	0,6885	1,0200	0,5631			0,6826
**5**	2,4667	0,5534	2,7867	0,3301			0,4483
**5e**	3,4267	0,6634	0,3867	0,3711			0,0053
**7**	4,4967	0,4274	1,8300	0,4321			0,0016
**14**	5,3100	0,3635	3,8033	0,2307			0,0063
**15**	6,4633	0,3584	4,1367	0,3510			0,0013
**15**	6,4633	0,3584			4,9166	0,1464	0,0023
**15**			4,1367	0,3510	9,9166	0,1464	0,0238
**17**	7,2533	0,1861	4,7600	0,2402			0,0001
**17**	7,2533	0,1861			7,5200	0,0964	0,0923
**17**			4,7600	0,2402	7,5200	0,0964	0,0001
