# Supplementary material for: Use of MSAP Markers to Analyse the Effects of Salt Stress on DNA Methylation in Rapeseed (Brassica napus var. oleifera)
Source: PLoS One. 2013 Sep 23;8(9):e75597. doi: 10.1371/journal.pone.0075597 (PMC3781078; doi:10.1371/journal.pone.0075597)
Supplement: Table S7 — Trehalose quantification in shoot samples of Exagone and root samples of Toccata. Analyses were carried out in triplicate. Average values and standard deviations (SD) are reported for each sample. T-Student test was applied to estimate the differences in trehalose accumulation between control and salt-stressed samples at 7 DAS and 14 DAS. (PDF) [file pone.0075597.s010.pdf]

Table S7. Trehalose quantification in shoot samples of Exagone and root samples of Toccata. Analyses were carried out in triplicate. Average values and standard deviations (SD) are reported for each sample. T-Student test was applied to estimate the differences in trehalose accumulation between control and salt-stressed samples at 7 DAS and 14 DAS.

Exagone					
sample	trehalose (µmol/g FW)	sample	trehalose (µmol/g FW)	sample	trehalose (µmol/g FW)
**4**	0,122	**7C**	0,136	**14C**	0,146
	0,135		0,143		0,145
	0,140		0,119		0,121
avg	0,1323	avg	0,1327		0,1373
SD	0,0093	SD	0,0123		0,0142
		**7S**	0,303	**14S**	0,315
			0,325		0,326
-			0,319		0,324
		avg	0,3157		0,3217
		SD	0,0114		0,0059
		t-Student	18,8862	t-Student	20,8420
		P-value	4,62915 e-05	P-value	3,13155 e-05

Toccata					
sample	trehalose (µmol/g FW)	sample	trehalose (µmol/g FW)	sample	trehalose (µmol/g FW)
**4**	0,107	**7C**	0,101	**14C**	0,125
	0,118		0,124		0,084
	0,099		0,095		0,095
avg	0,1080	avg	0,1067		0,1013
SD	0,0095	SD	0,0153		0,0212
		**7S**	0,112	**14S**	0,111
			0,093		0,077
-			0,1		0,098
		avg	0,1017		0,0953
		SD	0,0096		0,0172
		t-Student	0,4792	t-Student	0,3808
		P-value	0,6568	P-value	0,7227
